# Could the ketogenic diet induce a shift in thyroid function and support a metabolic advantage in healthy participants? A pilot randomized-controlled-crossover trial

**DOI:** 10.1371/journal.pone.0269440

**Published:** 2022-06-03

**Authors:** Stella Iacovides, Shane K. Maloney, Sindeep Bhana, Zareena Angamia, Rebecca M. Meiring

**Affiliations:** 1 Faculty of Health Sciences, Brain Function Research Group, School of Physiology, University of the Witwatersrand, Parktown, Johannesburg, South Africa; 2 School of Human Sciences, The University of Western Australia, Crawley, Australia; 3 Division of Endocrinology, Department of Endocrinology and Metabolism, Chris Hani Baragwanath Academic Hospital, University of the Witwatersrand, Parktown, Johannesburg, South Africa; 4 Faculty of Health Sciences, Movement Physiology Research Laboratory, School of Physiology, University of the Witwatersrand, Parktown, Johannesburg, South Africa; 5 Department of Exercise Sciences, University of Auckland, Newmarket, Auckland, New Zealand; PLOS, UNITED KINGDOM

## Abstract

**Background:**

The ketogenic diet (KD) has been shown to result in body mass loss in people with disease as well as healthy people, yet the effect of the KD on thyroid function and metabolism are unknown.

**Objective:**

We aimed to determine the effects of a KD, compared with an isocaloric high-carbohydrate low-fat (HCLF) diet, on resting metabolic rate and thyroid function in healthy individuals.

**Design:**

Eleven healthy, normal-weight participants (mean(SD) age: 30(9) years) completed this randomized crossover-controlled study. For a minimum of three weeks on each, participants followed two isocaloric diets: a HCLF diet (55%carbohydrate, 20%fat, 25%protein) and a KD (15%carbohydrate, 60%fat, 25% protein), with a one-week washout period in-between. Importantly, while on the KD, the participants were required to remain in a state of nutritional ketosis for three consecutive weeks. Crossover analyses and linear mixed models were used to assess effect of diet on body mass, thyroid function and resting metabolic rate.

**Results:**

Both dietary interventions resulted in significant body mass loss (p<0.05) however three weeks of sustained ketosis (KD) resulted in a greater loss of body mass (mean (95%CI): -2.9 (-3.5, -2.4) kg) than did three weeks on the HCLF diet (-0.4 (-1.0, 0.1) kg, p < 0.0001). Compared to pre-diet levels, the change in plasma T3 concentration was significantly different between the two diets (p = 0.003), such that plasma T3 concentration was significantly lower following the KD diet (4.1 (3.8, 4.4) pmol/L, p<0.0001) but not different following the HCLF diet (4.8 (4.5, 5.2) pmol/L, p = 0.171. There was a significant increase in T4 concentration from pre-diet levels following the KD diet (19.3 (17.8, 20.9) pmol/L, p < 0.0001), but not following the HCLF diet (17.3 (15.7, 18.8) pmol.L, p = 0.28). The magnitude of change in plasma T4 concentration was not different between the two diets (p = 0.4). There was no effect of diet on plasma thyroid stimulating hormone concentration (p = 0.27). There was a significantly greater T3:T4 ratio following the HCLF diet (0.41 (0.27, 0.55), p < 0.0001) compared to pre-diet levels but not following the KD diet (0.25 (0.12, 0.39), p = 0.80).

**Conclusions:**

Although the diets were isocaloric and physical activity and resting metabolic rate remained constant, the participants lost more mass after the KD than after the HCLF diet. The observed significant changes in triiodothyronine concentration suggest that unknown metabolic changes occur in nutritional ketosis, changes that warrant further investigation.

**Trial registration:**

Pan African Clinical Trial Registry: PACTR201707002406306 URL: https://pactr.samrc.ac.za/.

## Introduction

The most common reason that individuals report for initiating a ketogenic diet (KD) is a desire to lose weight [1]. Randomized controlled trials have reported that more body mass is lost when subjects follow a KD compared to a high-carbohydrate, low-fat (HCLF) diet [1–13]. In the absence of knowledge on the metabolic mechanisms that underpin the two diets, greater body mass loss on a KD has been attributed either to spontaneous reduction in energy intake [8,12,14] or reduced hunger and/or increased satiety. Reduced hunger or increased satiety is believed to result from the increased fat consumption [4,15–17], or to a direct effect of β-hydroxybutyrate (BOHB, the major circulating ketone body) acting as a satiety signal, and directly inhibiting appetite [18]. Investigations into the effect of a KD, compared with a conventional high-carbohydrate, low-fat (HCLF) diet, on metabolism, and in particular, on thyroid function and resting metabolic rate, are rare [2,14].

Thyroid hormones play a significant role in metabolism, and it is well-established that thyroid hormone status correlates directly with body mass and energy expenditure [19]. However, the effects of the KD on thyroid function are unclear [2,14]. Higher levels of thyroxine (T4) with no change in triiodothyronine (T3) have been reported in one study [14], while in another, ketosis resulted in lower levels of T3 [20]. Several other studies have reported reductions in T3 with carbohydrate restriction, but there are limitations to these studies as the majority had small samples sizes and perhaps, more importantly, measurements in those studies were done after only four [21,22] or seven [23] days of carbohydrate restriction. The utilization of ketone-bodies (accounting for 50–85% of oxidative metabolism) becomes significant only after three to seven days of starvation [24,25]. Hence, in these latter studies, the participants may not have reached nutritional ketosis, and so would not have had time to become “keto-adapted”; a state in which a coordinated set of metabolic adaptations have occurred that ensure appropriate inter-organ fuel supply given the reduced availability of glucose [26]. Nutritional ketosis, defined as circulating ketone levels higher than 0.5 mmol.*ℓ*^*-1*^, therefore may take several weeks to be achieved [26].

Investigations into the effect of a KD, compared with a conventional HCLF diet, on metabolism, and in particular, on thyroid function and resting metabolic rate are rare [2,14]. Most randomized-controlled trials (RCTs) have been done in overweight/obese populations, some with pre-existing chronic conditions such as insulin resistance, metabolic syndrome, or dyslipidaemia [27–35]; these can all influence thyroid function. Furthermore, most previous studies have not controlled for energy-intake (i.e. dietary interventions were not isocaloric), nor did they control for physical activity (energy output) [29,30,35].

In the study reported here, we aimed to determine the effect of a ketogenic diet on resting metabolic rate, and on thyroid function, in the absence of a variation in energy intake or energy expenditure in normal weight subjects who were free of disease. To achieve those ends, we used a randomized controlled-cross-over study in which healthy participants completed several weeks on a KD (with nutritional ketosis sustained for a minimum of three consecutive weeks) and several weeks on an isocaloric high carbohydrate—low fat (HCLF) diet. Throughout the study, the daily energy intake was based on the habitual intake of each individual. Similarly, energy expenditure was controlled by asking the participants to maintain physical activity at customary levels throughout the study.

## Subjects and methods

Ethical clearance was obtained from the University of the Witwatersrand’s Human Research Ethics Committee (Medical), which adheres to the principles of the Declaration of Helsinki (Clearance certificate no. M150849). All participants gave verbal and written consent. The study design of this RCT has been detailed elsewhere (S3) [36]. In brief, using standardized and customized screening questionnaires for sleep quality and general health, volunteers were screened to ensure that they were free from any chronic illness, depression, sleep disorders, for at least six months prior to the start of the study. Volunteers were excluded if they were overweight or obese (BMI ≥ 26 kg/m^2^), had any adverse cardiac or metabolic conditions such as type 1 or 2 diabetes mellitus, hypercholesterolemia, or hypertension confirmed by a clinician. The Pittsburgh Sleep Quality Index (PSQI) [37] and the General Health Questionnaire (GHQ) [38] were used to assess quality of sleep and psychological health, respectively. Eligible participants were asked to maintain the same level of physical activity throughout the study, which was monitored using the standardized global physical activity questionnaire (GPAQ) [39]. Thereafter eligible participants completed, in randomized order, two isocaloric dietary interventions (KD and a HCLF diet) for a minimum of three weeks on each diet, with a one-week washout (habitual diet) between the diets. At the start of the study, the participants were required to complete and record their habitual diet for one week. Those data allowed us to determine the average daily energy intake of each participant. For the two dietary interventions, the total energy consumed each day was based on the average daily energy intake during that initial week. Thus, the two diets were isocaloric, but varied in macronutrient content, with the KD comprising 15% carbohydrate, 60% fat and 25% protein, and the HCLF diet comprising 55% carbohydrate, 20% fat and 25% protein. Randomization of the order of the diets was done using the Microsoft Excel 2010 (Version 14.0) CHOOSE and RANDBETWEEN functions. These functions (= CHOOSE(RANDBETWEEN(1,2), “A”,”B”) allowed the PI to assign each new participant to one of the two diets for the first intervention.

Each participant was provided with an individualized meal plan with their customized respective macronutrient content for each dietary intervention. In addition, other informative material on macronutrients and the energy density of food, including lists of acceptable foods, was provided to each participant. Provided that the macronutrient ratio (55% carbohydrate, 20% fat and 25% protein) and the total caloric intake (according to individualized habitual intake) were adhered to, participants on the HCLF diet were free to consume most kinds of carbohydrate (including starchy carbohydrates such as rice and pasta), but were asked to refrain from eating sugar and sugary food and / or drinks. In contrast, during the KD intervention, carbohydrate consumption was not only very low (accounting for only 15% of dietary intake, whilst energy from fat was at 60%, and protein the remaining 25%), but participants were limited to specific kinds of carbohydrate-rich food, such as leafy green vegetables, lettuce, and broccoli. In addition to providing individualized meal plans, the PI educated each participant on the fundamentals of each dietary intervention and provided each participant with a foodlist and other resources that contained information on dietary macronutrient composition and calories, to assist each participant in making food choices of their preference, but in line with the dietary intervention. Further, to assist with participant compliance, the principal investigator (PI) was available at all times to advise and motivate participants. When participants came into the study already supplementing their diet with vitamins and/or minerals, they were requested to continue doing so. If participants were not taking any supplements, they were asked to refrain from doing so. The participants were asked to also maintain the same level of physical activity (habitual and structured) throughout the study. Physical activity was assessed by calculating a metabolic equivalent (MET) score from the validated global physical activity questionnaire (GPAQ) [39], which was completed at the end of each week throughout the study. The GPAQ assesses time spent in physical activities ranging from walking to vigorous intensity activity and creates a MET score (MET minutes per week) which accounts for both duration and type of physical activity–a higher MET score indicates more time spent in higher intensity activity.

Each day throughout the study (i.e. during the first week of data collection on the habitual-diet, during the two dietary interventions, and during the washout period), the participants kept a detailed account of their daily dietary consumption using a specialized, but commercially available, online application that quantified energy intake, MyFitnessPal (MyFitnessPal, Inc. Version 18.8.5, Under Armour). By connecting smart devices, the PI remotely monitored, and critically assessed the macronutrient composition and energy intake of each participant. At the end of each day, each participant received feedback from the PI; either positive / encouraging comments, or advice on how to best amend their diet for the purpose of the study.

An important requisite of the study was that, during the KD, the participants maintained a state of nutritional ketosis for a minimum of three consecutive weeks. To ensure that nutritional ketosis was reached and maintained, frequent measurement of blood levels of β-hydroxybutyrate (BOHB) were made using blood obtained via finger prick and a handheld β-ketone analyser (Freestyle Optium, Abbott Diabetes Care Ltd, United Kingdom). Levels of BOHB were measured before and after each dietary intervention, and during the KD. We defined nutritional ketosis as BOHB levels above 0.4 nM. Once nutritional ketosis was reached (usually within 6–10 days of starting the KD), BOHB levels were measured every second day to ensure that the nutritional ketosis was sustained. When a participant did not remain in a ketogenic state, the dietary intervention period was prolonged until a consecutive 3-week sustained ketogenic state was achieved. Ketosis was achieved on average 9 (5–19 (min-max) days after starting the KD), and was maintained for an average of 29 (23–56) days.

Before and after each dietary intervention (i.e. four times in total), after an overnight fast, the participants reported to the Movement Physiology Research Laboratory, housed in the School of Physiology at the University of the Witwatersrand’s Medical School. At each visit, anthropometric measurements were made, by the same PI, and a venous blood sample was obtained for the assessment of thyroid function. The level of thyroid stimulating hormone (TSH), free triiodothyronine (T3) and free thyroxine (T4) were measured by a reputable external laboratory (Clinical Laboratory Services, Johannesburg, South Africa).

After blood was taken, measurements of resting oxygen consumption and carbon dioxide production were made. From those measurements the respiratory exchange ratio (RER) was calculated, as an indication of the source of fuel that was being metabolised on each diet. Briefly, each participant lay down with a face mask covering their nose and mouth. The mask was attached to a metabolic cart (Quark Ergo, COSMED, Rome, Italy). The participants lay still for a period of 15 minutes and the last 5 minutes of oxygen consumption and carbon dioxide production was averaged and used for the calculation of RER. In addition, resting metabolic rate (RMR), expressd in energy terms, was calculated from oxygen consumption using the following formula [40]: ***RMR = Oxygen consumption (ℓ*.*min***^***-1***^***) x 4*.*8 kcal*.*ℓ***^***-1***^**. *The result was then multiplied by 60 to convert minutes to hours*, *and divided by body mass*.**

All outcomes were measured at baseline, and before and after three weeks of HCLF and three weeks of sustained nutritional ketosis. The study primary outcome of this study was diet-induced change in basal metabolic rate and thyroid function.

### Statistical analyses

To quantify the compliance of the participants on each diet, the prescribed total energy intake that was reported by each participant over the screening week was used to determine the proportion of macronutrients that were required within each diet. Each participant was required to achieve these pre-determined targets during the time on each interventional diet (KD and HCLF). Compliance for both energy and macronutrient (calories and grams of each macronutrient) were calculated as the percentage that the reported daily calories / grams of macronutrient consumption was of the daily target (as determined by individualized habitual caloric, and macronutrient composition of each dietary intervention), and are reported as mean (95% confidence interval). The STATA pkcross command was used to test for period, sequence, and carryover effects for body mass, thyroid hormones, and RMR prior to testing for diet effects. The sequence of the diets were denoted as “ab” if the individual received the HCLF diet first and “ba” if the individual received the KD diet first. Carryover effects were tested even though a washout period was included in the study design. Analysis of all variables indicated that there was no significant carryover effect and therefore the carryover variable was excluded from subsequent models. Linear mixed effects models were then performed for all variables, testing for the main effects of diet and period and the interaction between the two, and using participants as random effects. Analyses were first conducted on absolute variables measured after each period i.e. baseline, wasout, and each of the two diets. The change from baseline for outcomes was also analysed between each diet. The baseline value that was used depended on the sequence that the diets were consumed, i.e. baseline for sequence “ab” and washout for sequence “ba”. A linear mixed effects model was also used to test the difference in the change in outcome between the diets, using diet and period and the diet X period interaction as fixed effects and participant as random effects. The difference in physical activity level was assessed using a generalised linear mixed model with fixed effects being diet and period and participant being a random effect. All of the data were analysed using Stata IC (Version 15.1, StatsCorp LLC, Texas, USA) and a two-tailed probability of p < 0.05 was considered to be statistically significant. All data are expressed as mean (95% CI), unless otherwise stated.

A sample size calculation indicated that a total of 20 participants would provide 85% power to obtain a medium effect size for BMR (Cohen’s d effect size  =  0.3) [41].

## Results

**Fig 1:**
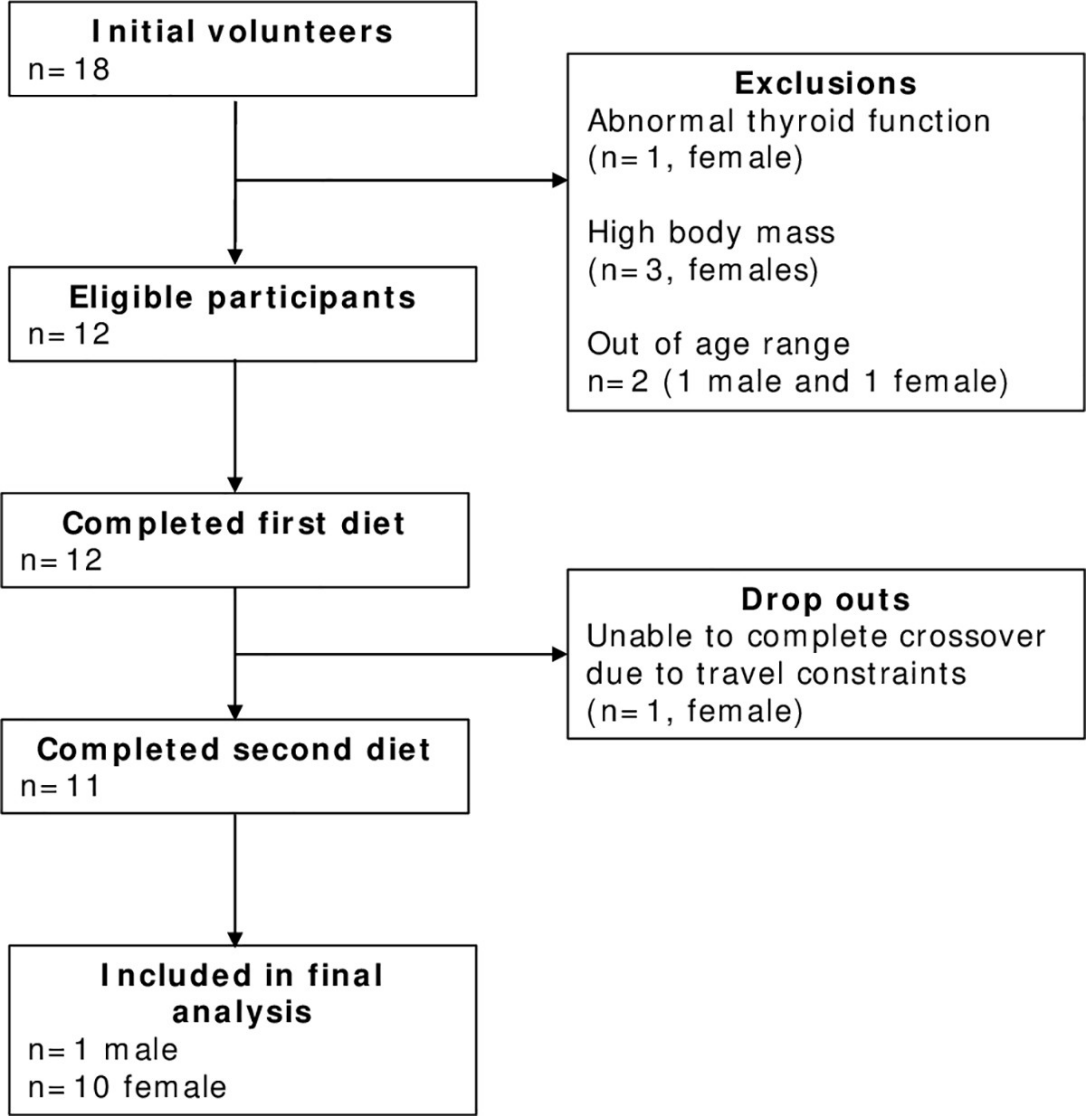
Flow of participants throughout the study from recruitment to inclusion in final analysis.

Eighteen individuals volunteered to take part in the study. One volunteer was excluded due to abnormal thyroid function, three others were excluded due to high BMI, two were excluded because of their advanced age with possible confounding factors due to menopause, and lastly, one volunteer was unable to report to the laboratory for regular testing and therefore did not finish the study. As displayed in Fig 1, eleven healthy male (n = 1) and female (n = 10) volunteers completed the study, with four participants completing the KD first. The demographic and screening tool results of the cohort are given in Table 1.

**Table 1:** Characteristics of the cohort prior to starting the first dietary intervention (n = 11).

Variable	Mean (SD)
Age (years)	30 (9)
Body mass (kg)	67 (12)
Height (m)	1.7 (0.1)
Body mass index (kg.m^-2^)	24 (2)
General Health Questionnaire^†^	3 (3)
Pittsburgh Sleep Quality Index^‡^	4 (1)

† Scores < 6 indicate good health [42].

‡ Scores ≤ 5 indicate good sleep quality [43].

### Dietary compliance and physical activity

Participants were adherent to the diets to within 96 (93, 99) % of the daily target of calories consumed for the HCLF diet and 96 (93, 98) % of the daily target of calories consumed for the KD (data are mean (95%CI)). Similarly, compliance for macronutrient consumption (grams of each macronutrient consumption over target proportions according to calories and dietary intervention), showed that participants were adherent to their targets within 93 (88, 98) % for carbohydrates, 87 (78, 97) % for fat, and 85 (76, 95) % for protein for the HCLF diet; and 76 (65, 86) % for carbohydrates, 91 (85, 97) % for fat, 91 (83, 99) % for protein for the KD diet. The dietary intake of carbohydrates on the KD had lowest compliance (76%), however, the mean daily intake was *lower* than the target intake, and this was likely the result of attempting to reach a state of ketosis.

Measurements of blood ketone level (BOHB) confirmed that during the KD the subjects were in a state of nutritional ketosis, and the respiratory exchange ratio (RER) suggested more fat oxidation on the KD, thus supporting a classification of nutritional ketosis (Table 2).

**Table 2:** Macronutrient energy intake, blood β-hydroxybutyrate (BOHB), and respiratory exchange ratio (RER) after the habitual diets of baseline and washout, and after the ketogenic diet (KD) and high-carbohydrate low-fat (HCLF) diet, for the whole cohort (n = 11).

Variable	Baseline	KD	Washout	HCLF
Total energy intake (cal)	1537.3 (534.3)	1471.5 (418.9)	1554.9 (473.9)	1456.0 (518.3)
Carbohydrates (% of total intake)	43.1 (4.6)	11.7 (3.2)	44.2 (5.3)	51.6 (4.0)
Fat (% of total intake)	36.9 (7.9)	64.3 (6.2)	37.2 (9.0)	23.8 (5.6)
Protein (% of total intake)	19.4 (4.3)	24.5 (3.2)	19.2 (4.5)	22.2 (2.1)
BOHB^†^	0.2 (0.2)	1.0 (0.5)	0.2 (0.1)	0.3 (0.2)
RER^‡^^*##*^	0.90 (0.10)	0.79 (0.05)	0.86 (0.04)	0.88 (0.07)
Physical Activity (MET score, min.wk^-1^)	1458 (510–3015)	1680 (1030–2283)	1280 (964–1365)	1995 (720–2467)

Data are represented as mean (SD) except for physical activity which is presented as median (IQR).

BOHB: β-hydroxybutyrate; RER: respiratory exchange ratio; RMR: resting metabolic rate.

† values > 0.4 indicate ketosis.

^‡^ values of 0.7 indicate that fat is the predominant fuel source; values of 0.85 suggests a mix of fat and carbohydrates; values of ≥ 1.00 indicate that carbohydrate is the predominant fuel source.

^##^data from n = 10.

Analysis of the MET scores (Table 2) from the GPAQ confirmed that physical activity levels remained the same throughout the study, and importantly between the dietary interventions (P = 0.57).

### Dietary effects on body mass and RMR

**Fig 2:**
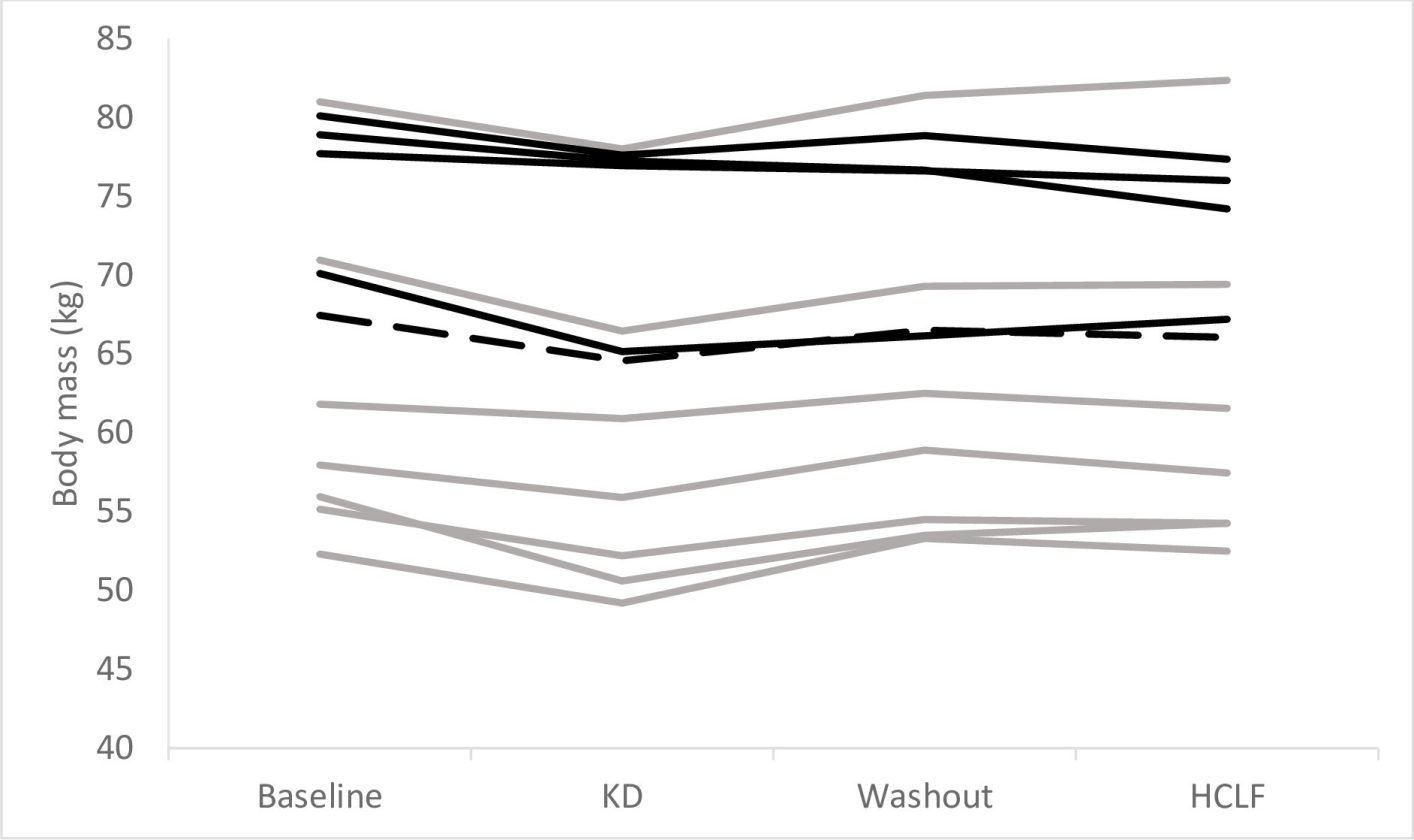
The individual changes in body mass (kg) during the dietary interventions. Solid black lines: participants who did the ketogenic diet (KD) diet first (‘ba’ intervention sequence); Grey lines: participants who did the high-carbohydrate low-fat (HCLF) diet first (‘ab’ intervention sequence); dashed line: mean for all participants.

Crossover analysis indicated that there was a significant diet (p = 0.0006) and period (p < 0.0001) effect on absolute body mass (Fig 2). Linear mixed effects analysis revealed that both of the diets resulted in a loss in body mass from baseline (Fig 2 and Table 3). The magnitude of the change in body mass on each of the diets was significantly greater on the KD diet (-2.9 (-3.5, -2.4) kg) compared to the HCLF diet (-0.4 (-1.0, 0.1) kg, mean (95%CI), p < 0.0001) (Table 3). There were no effects of diet on RMR (p > 0.05).

**Table 3:** Outcome measures of change in body mass and thyroid function between the two dietary interventions (n = 11).

Variable	KD	HCLF	Diet effect p value	Period effect p value
Change in body mass (kg)*	-2.9 (-3.5,-2.4)	-0.4 (-1.0, 0.1)	<0.0001	0.785
RMR (kcal.h^-1^.kg^-1^)^##^	5.53 (3.62,7.42)	5.08 (3.18,6.98)	0.082	0.016
Thyroid Stimulating Hormone (TSH, mIU/L)	2.06 (1.57,2.55)	2.32 (1.84,2.82)	0.071	0.042
Thyroxine (free T4, pmol.L^-1^)	19.3 (17.8, 20.9)	17.3 (15.7, 18.8)	0.0066	0.0004
Triiodothyronine (free T3, pmol.L^-1^)	4.1 (3.8, 4.4)	4.8 (4.5, 5.2)	<0.0001	0.0005
T3:T4 ratio	0.25 (0.12, 0.39)	0.41 (0.27, 0.55)	0.0467	0.0817

Data are presented as mean (95% CI).

All measures are within normal ranges: TSH (0.27–4.20 mIU/L), T4 (12.0–22.0 pmol/L), T3 (3.1–6.8 pmol/L).

KD, ketogenic diet; HCLF, high-carbohydrate low-fat diet.

*change from either baseline or washout according to diet sequence.

^##^data from n = 9.

### Dietary effects on thyroid hormones

**Fig 3:**
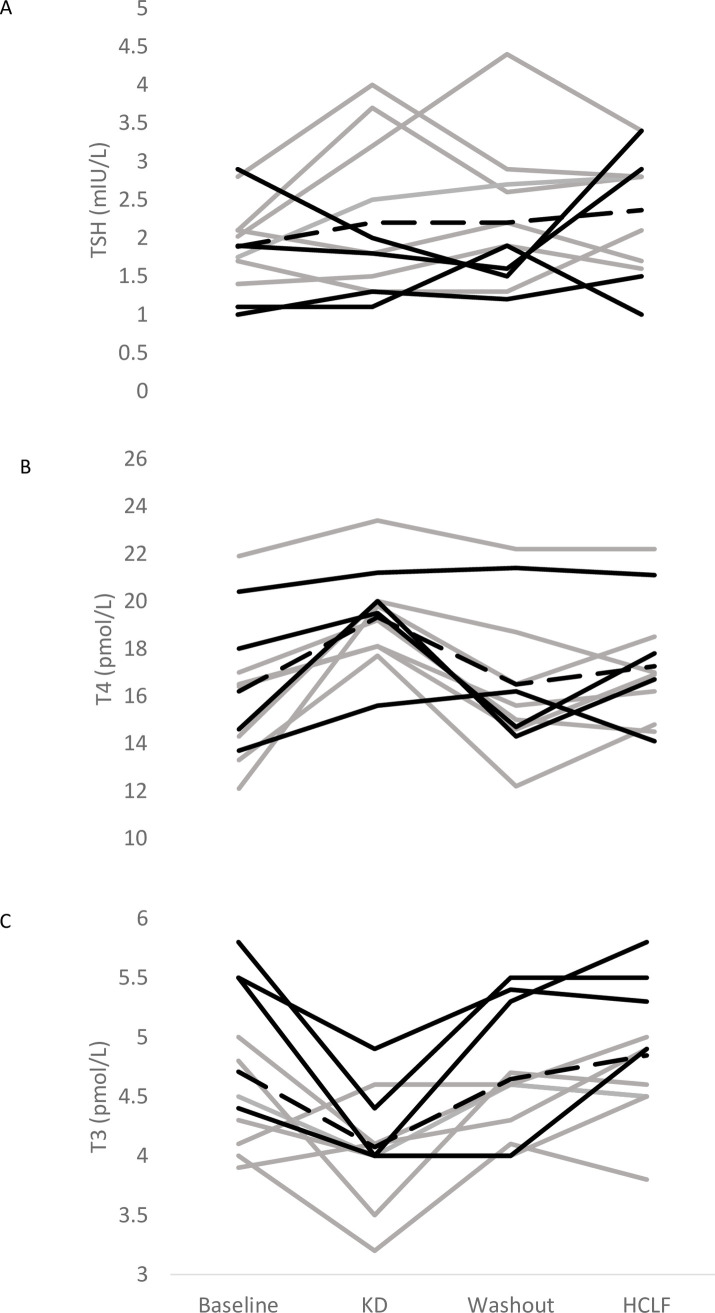
The individual changes in circulating levels of thyroid stimulating hormone (TSH) (panel A), free thyroxine (T4; panel B) and triiodothyronine (T3; panel C) during the dietary interventions. Solid black lines: participants who did the ketogenic diet (KD) diet first (‘ba’ intervention sequence); Grey lines: participants who did the high-carbohydrate low-fat (HCLF) diet first (‘ab’ intervention sequence); dashed line: mean for all participants.

Fig 3 shows the changes in absolute thyroid hormone concentrations throughout the study for all participants. Crossover analysis indicated a significant effect of diet (p < 0.0001), sequence (p = 0.02), and period (p < 0.0001) on absolute T3 concentration. There was also a significant effect of diet (p < 0.007) and period (p = 0.0004) on absolute T4 concentration. There was no diet (p = 0.27), period (p = 0.21), or sequence effect (p = 0.31) on TSH concentration (Table 3).

A linear mixed effects model showed that compared with pre-dietary intervention values (baseline / washout, depending on the sequence), T3 concentration was significantly lower following the KD diet (4.1 (3.8,4.4) pmol/L, p<0.0001) but it was not different following the HCLF diet (4.8 (4.5, 5.2) pmol/L, p = 0.171, Fig 3 and Table 3). The magnitude of change in T3 concentration following the KD diet (-0.66 (-1.21, -0.12) pmol/L) was also significantly different to that following the HCLF diet (0.60 (0.06, 1.14), pmol/L, p = 0.003). There was a significant increase in T4 concentration from baseline/washout following the KD diet (19.3 (17.8, 20.9) pmol/L, p < 0.0001), but not on the HCLF diet (17.3 (15.7, 18.8) pmol/L, p = 0.282, Fig 3 and Table 3). There was no difference in the magnitude of change in T4 concentration between the two diets (KD: 2.9 (1.6, 4.1), pmol/L; HCLF: 1.1 (-0.1, 2.3) pmol/L, p = 0.360). As such, although all thyroid hormone levels remained within the accepted “normal range”, the magnitude of change (from baseline/washout) was significantly different between the two diets was significant for T3, but not T4 concentration.

There was a significant effect of diet on T3:T4 ratio (p = 0.047) but no effect of sequence (p = 0.06) or period (p = 0.08). There was a significantly greater T3:T4 ratio following the HCLF diet (0.41 (0.27, 0.55), p < 0.0001) compared to baseline/washout but not following the KD diet (0.25 (0.12, 0.39), p = 0.80) compared to baseline/washout (Table 3).

## Discussion

In this randomized controlled-crossover study in healthy, normal-weight individuals, we found that, despite no difference in energy intake (diet) or energy expenditure (resting metabolic rate and physical activity) between the diets, three consecutive weeks in a state of sustained nutritional ketosis resulted in greater loss of body mass than did three weeks on an isocaloric HCLF diet. The greater loss of body mass occurred despite no change in the resting metabolic rate or physical activity level. Further, although plasma TSH and T4 remained unchanged after each diet, the plasma T3 concentration decreased more on the KD than it did on the HCLF diet. The level of all of the thyroid hormones remained within the normal range.

To our knowledge, this is the first RCT that has assessed the resting metabolic rate (RMR) of people on a KD. The maintenance of a healthy body mass, or the loss of body mass during dieting, is generally framed in terms of the balance between energy intake and energy expenditure. On the expenditure side, RMR is the largest contributor, and the least modifiable component [44], while exercise associated thermogenesis (EAT), non-exercise associated thermogenesis (NEAT), and the thermic effect of food (TEF) are more modifiable. Since NEAT is the largest of those three components, a major aspect of our experimental design was to reduce the confounding effect of changes in NEAT by monitoring physical activity and encouraging the participants to maintain their normal levels of NEAT and EAT. That intervention was especially important because metabolic compensation following weight-loss can result in a decrease in NEAT and a decrease in the motivation to exercise, which will lead to a decrease in EAT [44]. At the same time, our experimental design ensured that energy intake was constant at each participant’s habitual daily energy consumption. By controlling those factors we mitigated compensatory changes in metabolism, allowing us to determine whether dietary macronutrient composition alone (i.e. changes in the relative contribution of each macronutrient, without changing total energy intake) could affect metabolic rate, and related thyroid function.

Using thermodynamics to explain the results on each of the diets creates a conundrum. On the HCLF diet, the average energy intake was less than RMR, therefore if we assume that participants lost a mixture of fat (37 kJ.g^-1^) and protein (17 kJ.g^-1^), and assuming that about half of the body mass loss was accounted for by water, the body mass loss on that diet would be within the range that was observed in our study. On the KD, the energy intake was also less than RMR and using the same logic as above, participants should have lost 31 grams.day^-1^, or 641 grams in three weeks. However, participants lost close to 3 kg, with a range of results between a loss of 1.5 and 4.8 kg. Therefore, the normal thermodynamics of energy balance seems to *not* explain our results.

However, we are not the first to report such a phenomenon. Some [1–13,45], but not all [29,31,33–35,46–50] RCTs that have investigated changes in body mass during a KD compared to a HCLF diet have reported more body mass loss on the KD. The effects are ascribed to either a spontaneous reduction in energy intake because of increased satiety, or reduced hunger, that comes with increased fat consumption [4,15–17]. Comparisons to the present study are difficult, however, as many of the studies were conducted in people with chronic disease, many did *not* account for energy intake (i.e. dietary interventions were not isocaloric), and most did not measure physical activity or encourage similar energy expenditure across interventions. In addition, since energy intake and physical activity were the same on the two intervention diets in this study, those explanations for the change in body mass do not hold, for our study.

When someone on a low-carbohydrate diet loses body mass, it is often attributed to the water loss that accompanies a decrease in glycogen storage. But that water loss is always short-lived [2]. While some argue that a reduction in carbohydrate intake leads to the mobilization of adipose tissue, thus preserving lean body tissue [3,14], others argue that carbohydrate restriction may lead to the preferential loss of lean body tissue [51]. Further, it is argued that there is a “metabolic advantage” with the KD. For example, one study reported that in overweight adolescents, despite an increase in energy intake on the KD, the participants lost more body mass on the KD than on on the HCLF diet [13]. Accordingly, it is suggested that a non-restricted KD may be more effective for weight-loss than a low-energy diet due the maintenance of a higher metabolic rate [13]. Thus, high energy intake on a KD diet may ameliorate the metabolic responses to energy restriction that are observed on very-low-energy diets [13,52,53]. More studies, preferably done over longer periods (>1 year), are required to determine the effect of the KD versus a HCLF diet on metabolic rate, with and without a energy restriction.

A likely candidate for a “metabolic advantage” of the KD is a change in thyroid function. Although the complex interactions of the various pathways that involve thyroid hormones are still not fully understood, it is well recognized that thyroid hormones influence key metabolic pathways that are involved in energy balance (energy expenditure and storage), and that they are also active in lipid and carbohydrate metabolism. Hence, thyroid hormones influence metabolic rate and adaptive thermogenesis, and thus have a significant impact on body mass. Variations in thyroid hormone transporter expression, local ligand activation and inactivation, the relative expression of thyroid hormone receptor isoforms, as well as the activity of receptor corepressors and coactivators, can all impact on thyroid function [19]. For the purpose of this discussion, we will highlight only a few key mechanisms, and speculate on areas that might warrant investigation to provide insight into the phenomenon that we, and others, have described.

When thyrotropin releasing hormone (TRH) is released from the pituitary, it stimulates the thyroid to release thyroid hormone. About 80% of the secretion from the thyroid is T4, with about 20% T3 [54]. The affinity of the intracellular thyroid hormone receptor (TR) is about 10 times higher for T3 than for T4, and so thyroid activity is generally taken to be proportional to T3, the active form of the hormone [54]. Because metabolic rate and energy expenditure are related to thyroid activity, it might be expected that the lower T3 on the KD should have led to an energy excess on that diet. But the participants lost more body mass on the KD than on the HCLF diet. Clearly a simple interpretation, based on the circulating level of active hormone, is wrong.

Thyroid hormone exerts its action when it binds to TR that are intracellular, and the intracellular activity of thyroid hormone depends mainly on the intracellular, rather than the circulating, concentration of the hormone. Locally, T4 is converted to T3 by intracellular deiodinases. 5´-deiodinase type (D1) is situated in the plasma membrane. The T3 that is produced by D1 exits the cell within about 30 minutes and enters the plasma pool. D2 is situated on the endoplasmic reticulum, close to the nucleus where the TR are located, and T3 created by D2 remains in the cell for about 8 hours. D3 is also located in the plasma membrane, where it deiodinates both T3 and T4, inactivating them to T2. Thus, the activation of TR, and subsequent changes in gene expression with profound effects on metabolism, depends on the circulating levels of T3 and T4 as well as the local activity of D2 versus D1 and D3. The specific activity of the deodinases can be modified by several environmental factors [54], and so it is possible that the KD induced changes in the activity of D2 relative to D1 and D3 that resulted in the maintenance, or perhaps even an increase, in a component of energy expenditure that we did not measure, despite a reduction in T3.

The importance of signalling from the nutritional status of the cell is increasingly recognized as a contributor to the control of metabolic processes [19]. A promising target to explain the metabolic effects of the KD is the nuclear receptor corepressor (NCoR). At least in rodents, the expression of NCoR is reduced when they consume a high fat diet [19]. Further, NCoR knockouts develop obesity, and the knockout has tissue specific effects [55], with an increase in mitochondrial content, an increase in oxidative metabolism, and an increase in insulin sensitivity in skeletal muscle, but increased triglyceride synthesis and insulin sensitivity in adipose tissue [55]. Our participants all had a healthy BMI, meaning that any changes resulting from altered NCoR signalling would have been more noticeable in skeletal muscle than adipose tissue, a situation that would be markedly different in obese subjects. NCoR is recognized as an essential metabolic regulator [56], and more research is warranted to determine it’s role in metabolism during nutritional ketosis.

The Retinoid X Receptor (RXR), a member of the nuclear receptor superfamily, could also be worthy of further investigation. RXR knockout mice are resistant to weight gain when they are fed a high-fat diet, in part due to an increase in the activity of lipoprotein lipase in skeletal muscle [57]. Furthermore, bile acids are increasingly recognized as general metabolic integrators, and bile acid stimulating pathways are associated with a thyroid hormone-mediated increase in energy expenditure [19,58–60]. More research is required on bile acid synthesis, as well as, the receptor for bile acids [61] and their possible role in metabolic changes with the KD.

In a continuous state of nutritional ketosis, other endocrine adaptations are likely involved in processes such as lipogenesis, lipolysis, and protein synthesis and degradation. For example, infusions of BOHB reduce proteolysis during starvation [62]. However, it is likely that other anabolic hormones, such as growth hormone, work in conjunction with BOHB. In a state of nutritional ketosis, changes in cortisol, insulin, insulin-like growth factor-1, glucagon, testosterone, growth hormone, and catecholamines, could also play a role. In one of the rare studies that assessed an array of hormones following dietary intervention, no changes in cortisol, testosterone, glucagon, sex-hormone-binding globulin, insulin-like growth factor-1, or T3 uptake were found in healthy men of normal weight after six weeks on a low-carbohydrate diet [14]. However, insulin levels were significantly reduced, and as we observed in the current study, total serum T4 was increased [14]. Serum T3 levels were not reported in that study [14], but others have reported lower T3 levels following carbohydrate restriction [20–23]. An early study in endurance-trained cyclists, also reported lower T3 after two weeks on a KD, compared to a eucaloric balanced diet [20]. The authors speculate that the fall in T3 may be an adaptation that conserves muscle glycogen [20]. While there are variations in participant demographics and study designs, the general outcome seems to be that carbohydrate restriction, or nutritional ketosis, results in a shift in the ratio of circulating thyroid hormones, without a change in TSH, with an increase in circulating inactive T4, and a reduction in active T3. Our findings of a significantly higher fT3/fT4 after the HCLF dietary intervention compared with after the KD dietary intervention is in line with some [63–65], but not all [66] studies that have investigated associations between T3/T4 ratio with metabolic syndrome. Evidence suggesting that a higher ratio is associated with increased risk of metabolic syndrome and/or insulin resistance has been reported in obese Japanese boys with metabolic syndrome compared to boys without [63], European men and women [64] and in euthyroid middle-aged adults [65].

Although animal and human studies show that exposure to cold enhances the activity of brown adipose tissue (BAT) [67,68] which generates heat and helps to maintain body temperature, the role of BAT in diet induced thermogenesis and the maintainance of energy homeostasis remains controversial [69,70]. BAT is activated by insulin [67], but that activation is not the same as the activation by cold. In response to insulin, the glucose uptake by BAT increased 5-fold with no change in perfusion, whereas when BAT was activated by cold, glucose uptake increased 12-fold and perfusion doubled, and the perfusion of BAT was positively associated with whole-body energy expenditure [67]. We did not measure the metabolic activity in BAT in our participants. However, it is intriguing that the shift in thyroid hormones that we saw after KD were similar to the shift reported during cold exposure [68]. Extrapolation of these results might suggest that the metabolic adaptations that occur during nutritional ketosis, and a likely reduced insulin release, may be similar to metabolic activity that occurs in BAT during cold exposure. Other important metabolic factors, such as an increase in D2 activity [19,58,60], need to occur for thermogenesis in BAT. These possibilities together with reasons why metabolic rate was not higher in our KD participants, require further investigation before we can conclude that a KD induces an increase in metabolic activity in BAT.

The results of our study need to be viewed in the context of the study limitations, as well as the study strengths. One study limitation that needs to be considered is the sample size. We recruited 11 healthy, normal weight individuals who fulfilled our study criteria and who were willing to partake in a challenging randomized crossover design study protocol. Our sample size calculation (on RMR) indicated that total of 20 participants would have provided 85% power to obtain a medium effect size for RMR (Cohen’s d effect size = 0.3). Nevertheless, our sample size had sufficient power to detect changes in body mass and thyroid hormones during the dietary interventions, and we believe that our findings add important insight into the litertaure. Ideally, our protocol would be improved by; 1) a larger sample size, 2) a longer dietary intervention period, 3) obtaining objective physical activity data to ensure that NEAT remained the same throughout the study, and 4) a more equal representation of males to females (as opposed to a 1:10 ratio). Lastly, although statistical analyses confirmed no sequence effect, our study could have been strengthened had the numbers of participants beginning each diet been more balanced. In light of our discussion above, measurements of specific receptor activity, and / or D2 activity, and perhaps also T2 and reverse T3 [71] would also provide insight into the mechanisms underlying our results. Future studies should also look deeper into the ‘period’ effect that was evident in this pilot study. It is possible that with increasing time spent participating in studies involving dietary interventions, there is increased awareness of food choices, which over time, could lead to weight loss over time, regardless of dietary intervention.

This study design had the following strengths. Our participants were healthy, normal weight individuals who were free from chronic conditions, and the participants completed the two diets in a random order, such that they each acted as their own control. By frequently measuring blood ketone levels, we ensured that each participant remained in a state of nutritional ketosis for three consecutive weeks and, by measuring RER, we were able to determine the predominant fuel substrate. Although dietary adherence relied on honest subjective reporting, our objective physiological measures, such as BOHB and RER, confirmed the presence or absence of nutritional ketosis, and we found a participants were 96% complaint with the daily target of calories consumed for both diets. Further, to limit dietary regression we supplied customized meal plans according to the individual preferences of each participant, and daily energy per macronutrient, in addition to other educational sources containing information for matching alternative foods. We further requested daily recording of food intake, and the participants received daily feedback. Lastly, we ensured that physical activity was consistent across the study.

## Conclusions

This study has shown that despite the two diets (KD and HCLF) being isocaloric, and despite no difference in physical activity, a greater reduction in body mass occurred on the KD in healthy weight participants, despite no change in RMR. The change in concentration in thyroid hormones (particularly, active T3) suggests that nutritional ketosis may induce metabolic changes that warrant further investigation.

## Supporting information

S1 File This is the document for CONSORT checklist for RCTs. (DOC)

S2 File This is the SPIRIT Figure. (DOC)

S3 File This is the protocol that was published in Trials: Iacovides et al (2018). (PDF)

S4 File This is the full protocol for which ethics was approved. (DOCX)

S1 Raw data (ZIP)

## Abbreviations

KD: ketogenic diet
HCLF: high-carbohydrate low-fat
RCTs: randomized controlled trials
T3: triiodothyronine
T4: thyroxine
TSH: thyroid stimulating hormone
BOHB: β-hydroxybutyrate
BMI: body mass index
PI: principal investigator
MET: metabolic equivalent
GPAQ: global physical activity questionnaire (GPAQ)
RER: respiratory exchange ratio
RMR: resting metabolic rate
SD: standard deviation
RM-ANOVA: repeated-measures analysis of variance EAT, exercise associated thermogenesis
NEAT: non-exercise associate thermogenesis
TEF: thermic effect of food
TR: thyroid hormone receptor
NCoR: nuclear receptor corepressor
RXR: Retinoid X receptors
BAT: brown adipose tissue
D1: 5´-deiodinase type 1
D2: 5´-deiodinase type 2
D3: 5´-deiodinase type 3

## References

[1] Brehm BJ, Seeley RJ, Daniels SR, D’Alessio DA. A randomized trial comparing a very low carbohydrate diet and a calorie-restricted low fat diet on body weight and cardiovascular risk factors in healthy women. J Clin Endocrinol Metab 2003; 88:1617–23. doi: 10.1210/jc.2002-021480 12679447

[2] Yancy WS Jr., Olsen MK, Guyton JR, Bakst RP, Westman EC. A low-carbohydrate, ketogenic diet versus a low-fat diet to treat obesity and hyperlipidemia: a randomized, controlled trial. Ann Intern Med 2004; 140:769–77. doi: 10.7326/0003-4819-140-10-200405180-00006 15148063

[3] Volek J, Sharman M, Gomez A, Judelson D, Rubin M, Watson G, et al. Comparison of energy-restricted very low-carbohydrate and low-fat diets on weight loss and body composition in overweight men and women. Nutr Metab (Lond) 2004; 1:13. doi: 10.1186/1743-7075-1-13 15533250 PMC538279

[4] Nickols-Richardson SM, Coleman MD, Volpe JJ, Hosig KW. Perceived hunger is lower and weight loss is greater in overweight premenopausal women consuming a low-carbohydrate/high-protein vs high-carbohydrate/low-fat diet. J Am Diet Assoc 2005; 105:1433–7. doi: 10.1016/j.jada.2005.06.025 16129086

[5] Krauss RM, Blanche PJ, Rawlings RS, Fernstrom HS, Williams PT. Separate effects of reduced carbohydrate intake and weight loss on atherogenic dyslipidemia. Am J Clin Nutr 2006; 83:1025–31; quiz 205. doi: 10.1093/ajcn/83.5.1025 16685042

[6] McClernon FJ, Yancy WS Jr., Eberstein JA, Atkins RC, Westman EC. The effects of a low-carbohydrate ketogenic diet and a low-fat diet on mood, hunger, and other self-reported symptoms. Obesity (Silver Spring) 2007; 15:182–7. 17228046 10.1038/oby.2007.516

[7] Halyburton AK, Brinkworth GD, Wilson CJ, Noakes M, Buckley JD, Keogh JB, et al. Low- and high-carbohydrate weight-loss diets have similar effects on mood but not cognitive performance. Am J Clin Nutr 2007; 86:580–7. doi: 10.1093/ajcn/86.3.580 17823420

[8] Westman EC, Yancy WS Jr., Mavropoulos JC, Marquart M, McDuffie JR. The effect of a low-carbohydrate, ketogenic diet versus a low-glycemic index diet on glycemic control in type 2 diabetes mellitus. Nutr Metab (Lond) 2008; 5:36. doi: 10.1186/1743-7075-5-36 19099589 PMC2633336

[9] Shai I, Schwarzfuchs D, Henkin Y, Shahar DR, Witkow S, Greenberg I, et al. Weight loss with a low-carbohydrate, Mediterranean, or low-fat diet. N Engl J Med 2008; 359:229–41. doi: 10.1056/NEJMoa0708681 18635428

[10] Forsythe CE, Phinney SD, Fernandez ML, Quann EE, Wood RJ, Bibus DM, et al. Comparison of low fat and low carbohydrate diets on circulating fatty acid composition and markers of inflammation. Lipids 2008; 43:65–77. doi: 10.1007/s11745-007-3132-7 18046594

[11] Keogh JB, Brinkworth GD, Noakes M, Belobrajdic DP, Buckley JD, Clifton PM. Effects of weight loss from a very-low-carbohydrate diet on endothelial function and markers of cardiovascular disease risk in subjects with abdominal obesity. Am J Clin Nutr 2008; 87:567–76. doi: 10.1093/ajcn/87.3.567 18326593

[12] Volek JS, Phinney SD, Forsythe CE, Quann EE, Wood RJ, Puglisi MJ, et al. Carbohydrate restriction has a more favorable impact on the metabolic syndrome than a low fat diet. Lipids 2009; 44:297–309. doi: 10.1007/s11745-008-3274-2 19082851

[13] Sondike SB, Copperman N, Jacobson MS. Effects of a low-carbohydrate diet on weight loss and cardiovascular risk factor in overweight adolescents. J Pediatr 2003; 142:253–8. doi: 10.1067/mpd.2003.4 12640371

[14] Volek JS, Sharman MJ, Love DM, Avery NG, Gomez AL, Scheett TP, et al. Body composition and hormonal responses to a carbohydrate-restricted diet. Metabolism 2002; 51:864–70. doi: 10.1053/meta.2002.32037 12077732

[15] Johnston CS, Tjonn SL, Swan PD. High-protein, low-fat diets are effective for weight loss and favorably alter biomarkers in healthy adults. J Nutr 2004; 134:586–91. doi: 10.1093/jn/134.3.586 14988451

[16] Westerterp-Plantenga MS, Lejeune MP, Nijs I, van Ooijen M, Kovacs EM. High protein intake sustains weight maintenance after body weight loss in humans. Int J Obes Relat Metab Disord 2004; 28:57–64. doi: 10.1038/sj.ijo.0802461 14710168

[17] Volek JS, Gomez AL, Kraemer WJ. Fasting lipoprotein and postprandial triacylglycerol responses to a low-carbohydrate diet supplemented with n-3 fatty acids. J Am Coll Nutr 2000; 19:383–91. doi: 10.1080/07315724.2000.10718935 10872901

[18] Arase K, Fisler JS, Shargill NS, York DA, Bray GA. Intracerebroventricular infusions of 3-OHB and insulin in a rat model of dietary obesity. Am J Physiol 1988; 255:R974–81. doi: 10.1152/ajpregu.1988.255.6.R974 3059829

[19] Mullur R, Liu YY, Brent GA. Thyroid hormone regulation of metabolism. Physiol Rev 2014; 94:355–82. doi: 10.1152/physrev.00030.2013 24692351 PMC4044302

[20] Phinney SD, Bistrian BR, Evans WJ, Gervino E, Blackburn GL. The human metabolic response to chronic ketosis without caloric restriction: preservation of submaximal exercise capability with reduced carbohydrate oxidation. Metabolism 1983; 32:769–76. doi: 10.1016/0026-0495(83)90106-3 6865776

[21] Fery F, Bourdoux P, Christophe J, Balasse EO. Hormonal and metabolic changes induced by an isocaloric isoproteinic ketogenic diet in healthy subjects. Diabete Metab 1982; 8:299–305. 6761185

[22] Johannessen A, Hagen C, Galbo H. Prolactin, growth hormone, thyrotropin, 3,5,3’-triiodothyronine, and thyroxine responses to exercise after fat- and carbohydrate-enriched diet. J Clin Endocrinol Metab 1981; 52:56–61. doi: 10.1210/jcem-52-1-56 7005258

[23] Ullrich IH, Peters PJ, Albrink MJ. Effect of low-carbohydrate diets high in either fat or protein on thyroid function, plasma insulin, glucose, and triglycerides in healthy young adults. J Am Coll Nutr 1985; 4:451–9. doi: 10.1080/07315724.1985.10720087 3900181

[24] Garber AJ, Menzel PH, Boden G, Owen OE. Hepatic ketogenesis and gluconeogenesis in humans. J Clin Invest 1974; 54:981–9. doi: 10.1172/JCI107839 4430728 PMC301639

[25] Owen OE, Reichard GA Jr., Human forearm metabolism during progressive starvation. J Clin Invest 1971; 50:1536–45. doi: 10.1172/JCI106639 5090067 PMC292094

[26] Volek JS, Noakes T, Phinney SD. Rethinking fat as a fuel for endurance exercise. Eur J Sport Sci 2015; 15:13–20. doi: 10.1080/17461391.2014.959564 25275931

[27] Jonasson L, Guldbrand H, Lundberg AK, Nystrom FH. Advice to follow a low-carbohydrate diet has a favourable impact on low-grade inflammation in type 2 diabetes compared with advice to follow a low-fat diet. Ann Med 2014; 46:182–7. doi: 10.3109/07853890.2014.894286 24779961 PMC4025600

[28] Guldbrand H, Dizdar B, Bunjaku B, Lindstrom T, Bachrach-Lindstrom M, Fredrikson M, et al. In type 2 diabetes, randomisation to advice to follow a low-carbohydrate diet transiently improves glycaemic control compared with advice to follow a low-fat diet producing a similar weight loss. Diabetologia 2012; 55:2118–27. doi: 10.1007/s00125-012-2567-4 22562179 PMC3390696

[29] Yancy WS Jr., Westman EC, McDuffie JR, Grambow SC, Jeffreys AS, Bolton J, et al. A randomized trial of a low-carbohydrate diet vs orlistat plus a low-fat diet for weight loss. Arch Intern Med 2010; 170:136–45. doi: 10.1001/archinternmed.2009.492 20101008

[30] Krebs NF, Gao D, Gralla J, Collins JS, Johnson SL. Efficacy and safety of a high protein, low carbohydrate diet for weight loss in severely obese adolescents. J Pediatr 2010; 157:252–8. doi: 10.1016/j.jpeds.2010.02.010 20304413 PMC2892194

[31] Foster GD, Wyatt HR, Hill JO, Makris AP, Rosenbaum DL, Brill C, et al. Weight and metabolic outcomes after 2 years on a low-carbohydrate versus low-fat diet: a randomized trial. Ann Intern Med 2010; 153:147–57. doi: 10.7326/0003-4819-153-3-201008030-00005 20679559 PMC2949959

[32] Brinkworth GD, Buckley JD, Noakes M, Clifton PM, Wilson CJ. Long-term effects of a very low-carbohydrate diet and a low-fat diet on mood and cognitive function. Arch Intern Med 2009; 169:1873–80. doi: 10.1001/archinternmed.2009.329 19901139

[33] Deluis DA, Sagrado MG, Aller R, Izaola O, Conde R. Effects of C358A missense polymorphism of the degrading enzyme fatty acid amide hydrolase on weight loss, adipocytokines, and insulin resistance after 2 hypocaloric diets. Metabolism 2010; 59:1387–92. doi: 10.1016/j.metabol.2009.12.029 20102775

[34] Frisch S, Zittermann A, Berthold HK, Gotting C, Kuhn J, Kleesiek K, et al. A randomized controlled trial on the efficacy of carbohydrate-reduced or fat-reduced diets in patients attending a telemedically guided weight loss program. Cardiovasc Diabetol 2009; 8:36. doi: 10.1186/1475-2840-8-36 19615091 PMC2722581

[35] Hernandez TL, Sutherland JP, Wolfe P, Allian-Sauer M, Capell WH, Talley ND, et al. Lack of suppression of circulating free fatty acids and hypercholesterolemia during weight loss on a high-fat, low-carbohydrate diet. Am J Clin Nutr 2010; 91:578–85. doi: 10.3945/ajcn.2009.27909 20107198 PMC3132068

[36] Iacovides S, Meiring RM. The effect of a ketogenic diet versus a high-carbohydrate, low-fat diet on sleep, cognition, thyroid function, and cardiovascular health independent of weight loss: study protocol for a randomized controlled trial. Trials 2018; 19:62. doi: 10.1186/s13063-018-2462-5 29361967 PMC5782363

[37] Buysse DJ, Reynolds CF 3rd, Monk TH, Berman SR, Kupfer DJ. The Pittsburgh Sleep Quality Index: a new instrument for psychiatric practice and research. Psychiatry Res 1989; 28:193–213. doi: 10.1016/0165-1781(89)90047-4 2748771

[38] Goldberg DP, Rickels K, Downing R, Hesbacher P. A comparison of two psychiatric screening tests. Br J Psychiatry 1976; 129:61–7. doi: 10.1192/bjp.129.1.61 938806

[39] Bull FC, Maslin TS, Armstrong T. Global physical activity questionnaire (GPAQ): nine country reliability and validity study. J Phys Act Health 2009; 6:790–804. doi: 10.1123/jpah.6.6.790 20101923

[40] Melzer K. Carbohydrate and fat utilization during rest and physical activity. European e-Journal of clinical Nutrition and Metabolism 2011; 6:e45–52.

[41] Cohen J. A power primer. Psychol Bull 1992; 112:155–9. doi: 10.1037//0033-2909.112.1.155 19565683

[42] Goldberg DP, Rickels K, Downing R, Hesbacher P. A comparison of two psychiatric screening tests. The British Journal of Psychiatry 1976; 129:61–7. doi: 10.1192/bjp.129.1.61 938806

[43] Buysse DJ, Reynolds III CF, Monk TH, Berman SR, Kupfer DJ. The Pittsburgh Sleep Quality Index: a new instrument for psychiatric practice and research. Psychiatry research 1989; 28:193–213. doi: 10.1016/0165-1781(89)90047-4 2748771

[44] Trexler ET, Smith-Ryan AE, Norton LE. Metabolic adaptation to weight loss: implications for the athlete. J Int Soc Sports Nutr 2014; 11:7. doi: 10.1186/1550-2783-11-7 24571926 PMC3943438

[45] Gardner CD, Kiazand A, Alhassan S, Kim S, Stafford RS, Balise RR, Kraemer HC, King AC et al. Comparison of the Atkins, Zone, Ornish, and LEARN diets for change in weight and related risk factors among overweight premenopausal women: the A TO Z Weight Loss Study: a randomized trial. JAMA 2007; 297:969–77. doi: 10.1001/jama.297.9.969 17341711

[46] Cardillo S, Seshadri P, Iqbal N. The effects of a low-carbohydrate versus low-fat diet on adipocytokines in severely obese adults: three-year follow-up of a randomized trial. Eur Rev Med Pharmacol Sci 2006; 10:99–106. 16875041

[47] Morgan LM, Griffin BA, Millward DJ, DeLooy A, Fox KR, Baic S, Bonham MP, Wallace JM, MacDonald I, Taylor MA, et al. Comparison of the effects of four commercially available weight-loss programmes on lipid-based cardiovascular risk factors. Public Health Nutr 2009; 12:799–807. doi: 10.1017/S1368980008003236 18647427

[48] Tay J, Brinkworth GD, Noakes M, Keogh J, Clifton PM. Metabolic effects of weight loss on a very-low-carbohydrate diet compared with an isocaloric high-carbohydrate diet in abdominally obese subjects. J Am Coll Cardiol 2008; 51:59–67. doi: 10.1016/j.jacc.2007.08.050 18174038

[49] Sacks FM, Bray GA, Carey VJ, Smith SR, Ryan DH, Anton SD, McManus K, Champagne CM, Bishop LM, Laranjo N, et al. Comparison of weight-loss diets with different compositions of fat, protein, and carbohydrates. N Engl J Med 2009; 360:859–73. doi: 10.1056/NEJMoa0804748 19246357 PMC2763382

[50] Brinkworth GD, Noakes M, Buckley JD, Keogh JB, Clifton PM. Long-term effects of a very-low-carbohydrate weight loss diet compared with an isocaloric low-fat diet after 12 mo. Am J Clin Nutr 2009; 90:23–32. doi: 10.3945/ajcn.2008.27326 19439458

[51] Noakes M, Foster PR, Keogh JB, James AP, Mamo JC, Clifton PM. Comparison of isocaloric very low carbohydrate/high saturated fat and high carbohydrate/low saturated fat diets on body composition and cardiovascular risk. Nutr Metab (Lond) 2006; 3:7. doi: 10.1186/1743-7075-3-7 16403234 PMC1368980

[52] Feinman RD, Fine EJ. "A calorie is a calorie" violates the second law of thermodynamics. Nutr J 2004; 3:9. doi: 10.1186/1475-2891-3-9 15282028 PMC506782

[53] Feinman RD, Fine EJ. Thermodynamics and metabolic advantage of weight loss diets. Metab Syndr Relat Disord 2003; 1:209–19. doi: 10.1089/154041903322716688 18370664

[54] Abdalla SM, Bianco AC. Defending plasma T3 is a biological priority. Clin Endocrinol (Oxf) 2014; 81:633–41. doi: 10.1111/cen.12538 25040645 PMC4699302

[55] Li P, Fan W, Xu J, Lu M, Yamamoto H, Auwerx J, Sears DD, Talukdar S, Oh D, Chen A, et al. Adipocyte NCoR knockout decreases PPARgamma phosphorylation and enhances PPARgamma activity and insulin sensitivity. Cell 2011; 147:815–26. doi: 10.1016/j.cell.2011.09.050 22078880 PMC3783197

[56] You SH, Liao X, Weiss RE, Lazar MA. The interaction between nuclear receptor corepressor and histone deacetylase 3 regulates both positive and negative thyroid hormone action in vivo. Mol Endocrinol 2010; 24:1359–67. doi: 10.1210/me.2009-0501 20427468 PMC2903906

[57] Haugen BR, Jensen DR, Sharma V, Pulawa LK, Hays WR, Krezel W, Chambon P, Eckel RH et al. Retinoid X receptor gamma-deficient mice have increased skeletal muscle lipoprotein lipase activity and less weight gain when fed a high-fat diet. Endocrinology 2004; 145:3679–85. doi: 10.1210/en.2003-1401 15087432

[58] Gereben B, Zeold A, Dentice M, Salvatore D, Bianco AC. Activation and inactivation of thyroid hormone by deiodinases: local action with general consequences. Cell Mol Life Sci 2008; 65:570–90. doi: 10.1007/s00018-007-7396-0 17989921 PMC11131710

[59] Ockenga J, Valentini L, Schuetz T, Wohlgemuth F, Glaeser S, Omar A, Kasim E, duPlessis D, Featherstone K, Davis JR, et al. Plasma bile acids are associated with energy expenditure and thyroid function in humans. J Clin Endocrinol Metab 2012; 97:535–42. doi: 10.1210/jc.2011-2329 22162464

[60] Watanabe M, Houten SM, Mataki C, Christoffolete MA, Kim BW, Sato H, Messaddeq N, Harney JW, Ezaki O, Kodama T, et al. Bile acids induce energy expenditure by promoting intracellular thyroid hormone activation. Nature 2006; 439:484–9. doi: 10.1038/nature04330 16400329

[61] Svensson PA, Olsson M, Andersson-Assarsson JC, Taube M, Pereira MJ, Froguel P, Jacobson P et al. The TGR5 gene is expressed in human subcutaneous adipose tissue and is associated with obesity, weight loss and resting metabolic rate. Biochem Biophys Res Commun 2013; 433:563–6. doi: 10.1016/j.bbrc.2013.03.031 23523790 PMC3639367

[62] Sherwin RS, Hendler RG, Felig P. Effect of ketone infusions on amino acid and nitrogen metabolism in man. J Clin Invest 1975; 55:1382–90. doi: 10.1172/JCI108057 1133179 PMC301893

[63] Minami Y, Takaya R, Takitani K, Ishiro M, Okasora K, Niegawa T, Tamai H et al. Association of thyroid hormones with obesity and metabolic syndrome in Japanese children. J Clin Biochem Nutr 2015; 57:121–8. doi: 10.3164/jcbn.15-24 26388669 PMC4566020

[64] Wolffenbuttel BHR, Wouters H, Slagter SN, van Waateringe RP, Muller Kobold AC, van Vliet-Ostaptchouk JV, Links TP, van der Klauw MM et al. Thyroid function and metabolic syndrome in the population-based LifeLines cohort study. BMC Endocr Disord 2017; 17:65. doi: 10.1186/s12902-017-0215-1 29037214 PMC5644133

[65] Kim HJ, Bae JC, Park HK, Byun DW, Suh K, Yoo MH, Kim JH, Min YK, Kim SW, Chung JH et al. Triiodothyronine Levels Are Independently Associated with Metabolic Syndrome in Euthyroid Middle-Aged Subjects. Endocrinol Metab (Seoul) 2016; 31:311–9. doi: 10.3803/EnM.2016.31.2.311 27184017 PMC4923416

[66] Tarcin O, Abanonu GB, Yazici D, Tarcin O. Association of metabolic syndrome parameters with TT3 and FT3/FT4 ratio in obese Turkish population. Metab Syndr Relat Disord 2012; 10:137–42. doi: 10.1089/met.2011.0098 22229843

[67] Orava J, Nuutila P, Lidell ME, Oikonen V, Noponen T, Viljanen T, Scheinin M, Taittonen M, Niemi T, Enerback S, et al. Different metabolic responses of human brown adipose tissue to activation by cold and insulin. Cell Metab 2011; 14:272–9. doi: 10.1016/j.cmet.2011.06.012 21803297

[68] Din MU, Saari T, Raiko J, Kudomi N, Maurer SF, Lahesmaa M, Fromme T, Amri EZ, Klingenspor M, Solin O, et al. Postprandial Oxidative Metabolism of Human Brown Fat Indicates Thermogenesis. Cell Metab 2018; 28:207–16 e3. doi: 10.1016/j.cmet.2018.05.020 29909972

[69] Kozak LP. Brown fat and the myth of diet-induced thermogenesis. Cell Metab 2010; 11:263–7. doi: 10.1016/j.cmet.2010.03.009 20374958 PMC2867325

[70] Peterson CM, Orooji M, Johnson DN, Naraghi-Pour M, Ravussin E. Brown adipose tissue does not seem to mediate metabolic adaptation to overfeeding in men. Obesity (Silver Spring) 2017; 25:502–5 28117556 10.1002/oby.21721 PMC5323278

[71] Bianco AC, Salvatore D, Gereben B, Berry MJ, Larsen PR. Biochemistry, cellular and molecular biology, and physiological roles of the iodothyronine selenodeiodinases. Endocr Rev 2002; 23:38–89. doi: 10.1210/edrv.23.1.0455 11844744

