# Correction: Could the ketogenic diet induce a shift in thyroid function and support a metabolic advantage in healthy participants? A pilot randomized-controlled-crossover trial

**DOI:** 10.1371/journal.pone.0295112

**Published:** 2023-11-27

**Authors:** Stella Iacovides, Shane K. Maloney, Sindeep Bhana, Zareena Angamia, Rebecca M. Meiring

In Table 3, the outcome measures of change in body mass and thyroid function between the two dietary interventions (n = 11) were reported using joules instead of kcal as stated. Please see the correct Table 3 here.

**Table 3:** Outcome measures of change in body mass and thyroid function between the two dietary interventions (n = 11)

Variable	KD	HCLF	Diet effect p value	Period effect p value
Change in body mass (kg)*	-2.9 (-3.5,-2.4)	-0.4 (-1.0, 0.1)	<0.0001	0.785
RMR (kcal.h^-1^.kg^-1^)^##^	1.16 (1.01, 1.31)	1.14 (0.98, 1.29)	0.122	0.031
Thyroid Stimulating Hormone (TSH, mIU/L)	2.06 (1.57,2.55)	2.32 (1.84,2.82)	0.071	0.042
Thyroxine (free T4, pmol.L^-1^)	19.3 (17.8, 20.9)	17.3 (15.7, 18.8)	0.0066	0.0004
Triiodothyronine (free T3, pmol.L^-1^)	4.1 (3.8, 4.4)	4.8 (4.5, 5.2)	<0.0001	0.0005
T3:T4 ratio	0.25 (0.12, 0.39)	0.41 (0.27, 0.55)	0.0467	0.0817

Data are presented as mean (95% CI)

All measures are within normal ranges: TSH (0.27–4.20 mIU/L), T4 (12.0–22.0 pmol/L), T3 (3.1–6.8 pmol/L)

KD, ketogenic diet; HCLF, high-carbohydrate low-fat diet

*change from either baseline or washout according to diet sequence

^##^data from n = 9
