# Supplementary material for: Could the ketogenic diet induce a shift in thyroid function and support a metabolic advantage in healthy participants? A pilot randomized-controlled-crossover trial
Source: PLoS One. 2022 Jun 3;17(6):e0269440. doi: 10.1371/journal.pone.0269440 (PMC9165850; doi:10.1371/journal.pone.0269440)
Supplement: S2 File — (DOC) [file pone.0269440.s002.doc]

|  | **STUDY PERIOD** | | | | | | | |
| --- | --- | --- | --- | --- | --- | --- | --- | --- |
|  | **Enrolment** | **Allocation** | **Post-allocation** | | | | | **Close-out** |
| **TIMEPOINT**** | ***-6 months*** | **0**  **(1 Aug 2017)** | ***Habitual***  ***(1 wk)*** | ***Wk1*** | ***Wk2*** | ***Wk3*** | ***Washout***  ***(1 wk)*** | ***After completion of both diets (7 wk+ from baseline)*** |
| **ENROLMENT:** |  |  |  |  |  |  |  |  |
| **Eligibility screen** | X |  |  |  |  |  |  |  |
| **Informed consent** | X |  |  |  |  |  |  |  |
| **Allocation (and habitual dietary monitoring)** |  | X | X |  |  |  |  |  |
| **INTERVENTIONS:** |  |  |  |  |  |  |  |  |
| ***[HCLF]*** |  |  | X |  |  |  | X |  |
| ***[KD]*** |  |  | X |  |  |  | X |  |
| ***[List other study groups]*** |  |  |  |  |  |  |  |  |
| **ASSESSMENTS:** |  |  |  |  |  |  |  |  |
| ***BASELINE***  ***Sleep quality, mood cognitive test, physical activity.*** |  | X |  |  |  |  |  |  |
| ***OUTCOMES***  ***Outcome data collected every week during intervention:***  ***Sleep quality, physical activity.*** |  |  |  | x | x | x |  | X |
| ***Outcome data collected at the end of each intervention:***  ***Cognitive function, sleep quality, mood.*** |  |  |  |  |  |  | X | X |
|  |  |  |  |  |  |  |  |  |
| ***OTHER DATA COLLECTED DURING HABITUAL WEEK***  ***Cognitive practice tests, habitual dietary monitoring, general health*** |  |  | X |  |  |  |  |  |

S2_Figure 1. SPIRIT schedule of enrolment, interventions, and assessments for the duration of the study.

HCLF = High carbohydrate, low fat diet; KD= ketogenic diet, BMR = basal metabolic rate.
