# Supplementary material for: Could the ketogenic diet induce a shift in thyroid function and support a metabolic advantage in healthy participants? A pilot randomized-controlled-crossover trial
Source: PLoS One. 2022 Jun 3;17(6):e0269440. doi: 10.1371/journal.pone.0269440 (PMC9165850; doi:10.1371/journal.pone.0269440)
Supplement: S4 File — (DOCX) [file pone.0269440.s004.docx]

THE EFFECT OF A HIGH-FAT, LOW CARBOHYDRATE DIET ON PAIN, SLEEP AND COGNITIVE AND THYROID FUNCTION: A RANDOMISED CONTROLLED CROSS-OVER TRIAL

Contents:

1. Introduction
2. Study Objectives
3. Methods and Materials
   1. . Study design
   2. . Participants
   3. . Dietary Interventions
   4. . Dietary Monitoring
   5. . Assessments
   6. . Data analysis
4. Funding
5. Problems
6. References
7. **Introduction:**

In recent years a diet high in dietary fat and low in carbohydrates (CHO) has become a popular choice for people attempting to lose weight in South Africa. This is due to claims that high-fat, low carbohydrate (HFLC) eating can not only reduce weight but also improve health in every aspect including; improved blood glucose and insulin levels, increased mental focus and better sleeping habits [[1](#_ENREF_1)]. In addition, HFLC diets, otherwise known as ketogenic diets, have also been a source of scientific interest for many years in the treatment of certain medical conditions including epilepsy and neurodegenerative disorders [[2](#_ENREF_2)]. As a result, HFLC diets have become the subject of much debate among academics and lay persons alike.

According to nutritional guidelines [[3](#_ENREF_3)], a healthy balanced diet is considered to be high in CHO (45-65%), low in dietary fat (20-35%) with a moderate protein (10-35%) intake. However, HFLC diets promote reducing CHO intake to less than 50 grams per day and increasing healthy dietary fat to compensate for the energy deficit [[1](#_ENREF_1)]. Under normal feeding circumstances β-oxidation of fatty acids produces acetyl CoA which is then oxidized to carbon dioxide and water in Krebs cycle. However, when there is excessive amounts of acetyl CoA being produced, such as in fasting or in very low carbohydrate intake, nutritional ketosis results. This is a metabolic state where the liver converts acetyl CoA into ketones including: acetoacetate and 3-hydroxybutyrate which can then be utilized as energy in extra-hepatic tissues [[4](#_ENREF_4)].

Many studies [[5-10](#_ENREF_5)], but not all [[11-13](#_ENREF_11)] have shown that HFLC diets lead to greater weight loss in comparison to conventional low-fat, high carbohydrate (LFHC) diets. The biggest concern in using a HFLC diet for weight loss is the effect it has on cardiovascular health. Although HFLC diets have been shown to constantly increase high density lipoprotein (HDL) and decrease triglycerides [[11](#_ENREF_11), [12](#_ENREF_12), [14](#_ENREF_14), [15](#_ENREF_15)], the reported effects on low density lipoprotein (LDL) cholesterol are less consistent. While the results of some studies show increased LDL [[6](#_ENREF_6), [16](#_ENREF_16)], others report no change [[11](#_ENREF_11), [12](#_ENREF_12), [14](#_ENREF_14), [15](#_ENREF_15)] in LDL cholesterol. Few studies have analysed LDL particle size after HFLC diets and have found a shift from small dense LDL particles to large LDL particles, which are less atherogenic than the small particles [[15](#_ENREF_15), [17](#_ENREF_17), [18](#_ENREF_18)].

Another cardiovascular risk factor is elevated C-reactive protein (CRP) which is an acute phase protein used to measure of chronic inflammation [[19](#_ENREF_19)]. Results from studies that have investigated CRP levels and HFLC dietary intervention, are inconsistent, with some showing no change [[6](#_ENREF_6), [18](#_ENREF_18)] and others showing decreased CRP levels associated with weight loss [[16](#_ENREF_16), [20](#_ENREF_20), [21](#_ENREF_21)]. Very few studies have measured other inflammatory markers such as, interleukin 6 (IL-6), tumor necrosis factor alpha (TNF-α) and also show a decrease that is associated with weight loss [[21](#_ENREF_21)]. More research is required to clarify the link between HFLC diet and inflammatory mediators.

Furthermore, the relationship between pain and HFLC diets is not well understood. One study aimed to determine the direct effects of HFLC metabolism on pain and inflammation in a rat population. The rats were either fed a normal diet or a HFLC diet containing 79% fat for 3-4 weeks. Hindpaw withdrawal from thermal nociception was used as an objective measure of pain and hindpaw swelling and plasma extravasation as a measure of inflammation. The HFLC diet was found to have anti-inflammatory and hypoalgesic affects which were more pronounced in juvenile rats [[22](#_ENREF_22)]. Very little research on the link between pain and HCLF diets has been conducted in a human population. However, in two recent studies, HFLC diets were shown to positively affect migraine suffers. In a study of 108 migraine sufferers, who were randomised to either follow a HFLC diet or a standard low-calories diet for one month, frequency of migraines and drug consumption decreased in the HFLC group only [[23](#_ENREF_23)]. A similar reduction in frequency of migraines as well as a decrease in duration was shown with 15 migraine suffers during nutritional ketosis [[24](#_ENREF_24)]. In addition, higher levels of blood high-sensitivity-CRP have also been shown to negatively influence pain perception and function, and may result in sensitisation of peripheral nociceptors, ultimately contributing to development of a chronic pain state [[19](#_ENREF_19)]. This was illustrated in a study of 198 female twins where higher levels of CRP were associated with higher sensitivity to pain [[25](#_ENREF_25)].

Ketogenic diets have, in fact, been used to effectively treat epilepsy, and may have a similar anticonvulsant action to anticonvulsant drugs [[26](#_ENREF_26)]. A common link exists between the underlying biology of chronic pain and seizures as both involve increased excitability of neurons [[27](#_ENREF_27)]. In addition, neuropathic pain can be effectively treated using anticonvulsant drugs, which is often more effective than analgesics. As well as, decreased glycolytic metabolism (by any method) and blocking glycolysis with 2-deoxyglucose has been shown to have an anticonvulsant and analgesic effect [[27](#_ENREF_27)]. Adenosine also appears to have an influence on pain and adenosine signalling is increased by metabolic change such as fasting [[27](#_ENREF_27)]. Despite the many links between HFLC diets and pain and inflammation, very few studies have examined the direct effects in a healthy human population.

Although there is some evidence of a neuroprotective effect of HFLC diets in a range of brain disorders which are characterized by neuronal death, including Alzheimer’s disease, traumatic brain injuries and Parkinson’s disease [[2](#_ENREF_2)], only a few studies have evaluated cognitive performance in a normal human population. Rat studies have showed detrimental effects on the developing brains of rats fed a ketogenic diet, with significant impairments in visual-spatial learning and memory as well as decreased brain growth [[28](#_ENREF_28), [29](#_ENREF_29)]. In a human study of 93 overweight participants working memory and speed of processing after high- or low-fat diet consumption for 8 weeks was assessed, and results showed no difference between the groups in working memory but there was less of an improvement in speed of processing in the HFLC group [[30](#_ENREF_30)]. Further, in a recent study, 16 healthy men who consumed a HFLC diet for 5 days were also found to have impaired cognitive function with regard to attention, mood and speed [[31](#_ENREF_31)]. It must be noted, however, that 5 days on a HFLC diet is too short a period for these subjects to have reached a ketogenic state. The effects of a HFLC diet on cognition in a healthy population requires further study.

The psychological effect of ketogenic diets is also not well-established. One study on mood, hunger and other self-reported symptoms, including fatigue, somatic symptoms, insomnia and stomach problems in 119 overweight individuals over a six month period, showed less negative affect and hunger in the HFLC group compared with the group on a low-fat diet, and improvements in all other measures in both groups [[5](#_ENREF_5)]. Another study showed similar improvements in mood were found in both low- and high- carbohydrate weight-loss diets [[30](#_ENREF_30)].

Despite claims that HFLC diets can improve sleep, very little work has been done to assess this directly. In one study, six obese adolescents, who at baseline, had decreased rapid eye movement (REM) and excessive slow wave sleep (SWS), participated in a 20 week HFLC diet and showed improvements to near normal range in both sleep stages [[32](#_ENREF_32)]. Objective polysomnography was also used to evaluate the effect of a HFLC diet on sleep structure in eighteen children with therapy-resistant epilepsy. Results showed that a ketogenic diet improved sleep quality, decreased total sleep and increased REM sleep, whilst SWS was preserved [[33](#_ENREF_33)]. These changes were also considered to be a normalisation of sleep structure in this population as there is pathological percentage decrease of REM in epileptic children. A study of fourteen healthy, non-obese men who were good sleepers compared the acute effect of a HFLC to a LFHC diet, over a 48 hour period using polysomnographic testing. A significant increase in the percentage of SWS and reduced REM sleep as compared to total sleep time was observed in the HFLC diet group [[34](#_ENREF_34)]. Reduced total sleep time was also reported in overweight participants following a HFLC diet in another study using the Atkins Health Indicator test, although the difference was not significant as compared to the low fat diet group [[5](#_ENREF_5)]. The association between HFLC diet and sleep requires further research, particularly in a healthy population.

The associations between nutrition, pain, sleep and inflammation are clearly complex and largely not well-understood. Though they are clearly all interlinked. A National Health and Nutrition Examination Survey (USA) involving 9317 participants, for example, conducted found an association between higher levels of CRP and sleep disorders and poor quality of sleep [[35](#_ENREF_35)]. Earlier we discussed some literature suggesting an association between inflammation and pain, and inflammation and nutrition, and nutrition and sleep. This survey provides some evidence of an possible association between inflammation and sleep, and the link between pain and sleep is well-established [[36](#_ENREF_36)].

In addition, the effect of HFLC diet on thyroid function remains unclear. In a study of 20 normal weight men a 6 week HFLC diet increased total thyroxine (T_4_) and free T_4_ uptake but had no effect on triiodothyronine (T_3_) [[37](#_ENREF_37)]. In contrast, another study reported decreased T_3_ and no change in T_4_ following four days of CHO restriction in six healthy individuals [[38](#_ENREF_38)]. In another study of 120 overweight, hyperlipidemic individuals over a 24-week period, no difference was found in thyroid stimulating hormone (TSH) between the HFLC diet group and low-fat diet group [[39](#_ENREF_39)]. Further research is needed to clarify the effect of HFLC diets on thyroid function.

Therefore this study will aim to assess the effects of a HFLC diet compared to a low-fat diet on measures of pain, sleep, inflammation and cognitive and thyroid function in a healthy adult population.

1. **Study Objectives:**

The aim of this study is to investigate the effect of a HFLC diet compared to a LFHC diet on measures of a) muscle pain perception, b) sleep, c) inflammation, d) cognitive function and e) thyroid function.

Specific aims:

1. To determine the effect of a HFLC diet compared to a LFHC diet on muscle pain perception following a) exercise-induced delayed onset muscle soreness (DOMS) and b) experimentally-induced muscle pain using an injection of hypertonic saline into the muscle with DOMS.
2. To determine the effect of a HFLC diet compared to a LFHC diet on subjective measures of sleep.
3. To determine the effect of a HFLC diet compared to a LFHC diet on markers of inflammation including; Interleukin-6 (IL-6), Interleukin-1 (IL-1) high-sensitivity C-reactive protein (hsCRP), tumour necrosis factor alpha (TNF- α), ferritin and serum amyloid P (SAP).
4. To determine the effect of a HFLC diet compared to a LFHC diet on cognitive function using a battery of standardised cognitive tests.
5. To determine the effect of a HFLC diet compared to a LFHC diet on thyroid function by measuring thyroid stimulating hormone (TSH), triiodothyronine (T3), thyroxine (T4).

The hypothesis is that a 6-week period of HFLC dietary consumption will have positive benefits on measures of pain perception, sleep and inflammatory markers and may have a negative effect on cognitive function and thyroid function, compared to 6 weeks of LFHC dietary consumption.

1. **Methods and Materials:**

**3.1 Study design**

As outlined in Figure 1, participants will be asked to visit the Movement Physiology Research Laboratory on five occasions; the first visit will be for screening the volunteers to ensure that they fit the study-criteria. Thereafter, eligible participants will return to the laboratory before the start of and after each dietary intervention. Following one week of normal habitual dietary monitoring, each participant will follow two isocaloric diets (a ketogenic diet (KD) and a high-carbohydrate, low-fat (HCLF) diet) in a randomized order and separated by a one week washout period. . Randomization of the order of the diets will be done using the Microsoft Excel 2010 (Version 14.0) CHOOSE and RANDBETWEEN functions. These functions (=CHOOSE(RANDBETWEEN(1,2), “A”,”B”) are able to assign each new participant into one of the two diets. Each dietary intervention will last for a minimum period of three weeks. In the KD it is critical for the participant to remain in a ketogenic state for three consecutive weeks. Should the participant leave the ketogenic state (as assessed by blood ketone levels), the dietary intervention period will be prolonged until the consecutive 3-week ketogenic state is achieved. The principal investigator (PI) will not be blinded to the diet so that feedback may be provided on a daily basis, to encourage dietary compliance. A researcher that will evaluate the outcomes of the diet will however be blinded to the order in which the diets were undertaken.

**
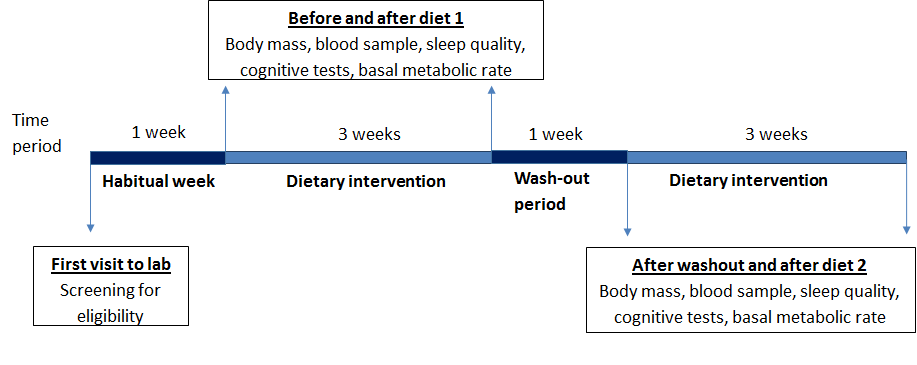
Figure 1.** Timeline and details of study procedures

**3.2 Participants**

Twenty healthy individuals, between the ages of 18 and 50 years, will be asked to volunteer in this study. A total of 20 participants would provide 85% power to obtain a medium effect size for BMR (Cohen’s d effect size = 0.3) [[40](#_ENREF_40)]. Using standardized and customized screening questionnaires for sleep quality and general health, volunteers will be screened to ensure that they are free from any chronic illness, depression, sleep disorders, for at least six months prior to the start of the study. Volunteers will be excluded if they are obese (BMI > 26 kg/m^2^), have any adverse cardiac or metabolic conditions such as type 1 or 2 diabetes mellitus, hypercholesterolemia or hypertension confirmed by a clinician. The Pittsburgh Sleep Quality Index (PSQI) [[41](#_ENREF_41)] and the General Health Questionnaire (GHQ) [[42](#_ENREF_42)] will be used to assess quality of sleep and psychological health, respectively. Eligible participants will be asked to maintain the same level of physical activity throughout the study, which will be monitored using the standardized global physical activity questionnaire (GPAQ) [[43](#_ENREF_43)]. If participants are taking a daily vitamin/mineral supplement, they will be asked to continue taking it as usual. If they are not taking any supplement they will be asked not to start taking one during the study. Using the dietary data obtained from one week of habitual dietary monitoring at the start of the study, daily calorie intake will be calculated for each participant and the total amount of calories consumed daily during both dietary interventions will be based on this value.

**3.3 Dietary intervention**

Participants will, in a randomized order, be allocated to follow a KD (15% carbohydrate, 60% fat and 25% protein) and an isocaloric HCLF diet (55% carbohydrate, 20% fat and 25% protein). Each dietary intervention will last for a minimum of three weeks, with a one week washout period in between each diet, during which time participants will be asked to follow their habitual diet (as during the first week of the study). The PI will provide each participant with meal plans that are specific to each dietary intervention, but will maintain an identical calorie intake to that which was consumed, on average, over the first week of following each participant’s habitual diet. Therefore the PI will formulate individualized meal plans with the correct respective macronutrient content for each dietary intervention according to each participant’s habitual caloric intake. The PI will also educate each participant on the fundamentals of each dietary intervention and provide foodlists and other resources containing information on dietary macronutrient compositions and calories, to assist each participant to make food choices of their preference, but in-line with the dietary intervention. The PI will also be easily assessable to each participant for guidance and/or advice, as well as motivation, throughout the study. Ultimately, these measures should assist with participant compliance.

**3.4 Dietary monitoring**

Throughout the study (i.e. during the first week (habitual week), during both dietary interventions and during the washout period), participants will be asked to record a detailed account of their daily dietary consumption using specialized but commercially available calorie counter applications. The PI will be able to remotely monitor (smart devices will be connected) and critically analyse and assess macronutrient composition and caloric intake, in order to provide each participant with feedback during the diets. The feedback provided will be either positive and encouraging comments, or advise on how to best amend their diet for the purpose of the study. In order to ensure that a nutritional ketosis is maintained for three consecutive weeks of being on the KD, blood levels of β-hydroxybutyrate (BOHB) will be determined using a finger prick test and a β-ketone handheld analyser (Freestyle Optium, Abbott Diabetes Care Ltd, United Kingdom). Levels of BOHB will be measured before and after each dietary intervention, as well as at the end of each week while participants are on the KD. This will be done to ensure that once the ketogenic state is reached, each participant stays in a ketogenic state for three consecutive weeks. Maintenance of a ketogenic state for three consecutive weeks will be a critical requirement of the study. When participants leave the ketogenic state (considered as BOHB levels below 0.4 nM), the dietary intervention period will be prolonged until the consecutive 3-week ketogenic state is achieved.

**3.5 Assessments**

*Home assessments*

During each dietary intervention, in addition to daily dietary recall, participants will be provided with study-specific morning and evening questionnaires to assess daily subjective measures of sleep, mood and exercise activity. Further, at the end of each week, physical activity will be assessed [[43](#_ENREF_43)].

*Sleep, mood and exercise questionnaires*

For the duration of the study period, participants will be required to complete a customized sleep diary every morning (morning questionnaire) to assess perceived sleep quality as well as morning vigilance on two separate 100-mm visual analogue scales (VAS), anchored from the “worst sleep” to the “best sleep ever”, and “not at all fresh and alert” to the “most fresh and alert ever”, respectively. The morning questionnaire will also provide information on each participant’s sleeping habits, such as what time they went to bed, how long they think it took them to fall asleep, and what time they woke up. Thus, information on each participant’s sleep-wake cycles can be retrieved. Each evening, throughout the study, participants will be required to complete a customized evening questionnaire to assess current mood on a 100-mm VAS anchored from “worst mood ever” to “best mood ever”. The evening questionnaire also asks for information on the type, duration and intensity of any exercise performed during the day. At the end of each week, physical activity will be further assessed using the standardized global physical activity questionnaire (GPAQ)[[43](#_ENREF_43)].

*Laboratory Assessments*

Before and after each dietary intervention (i.e. four times), participants will return to the laboratory after an overnight fast, for anthropometric measurements and to provide a blood sample. Tests to assess cognitive function tests and basal metabolic rate will also be assessed at each of these visits, and validated and standardized Pittsburgh Sleep Quality Index (PSQI)[[41](#_ENREF_41)] questionnaires will be administered to assess sleep quality.

*Cognitive function*

Cognitive function will be assessed using a battery of computerized cognitive tests (Cogstate computerized tests ([www.cogstate.com](http://www.cogstate.com))) performed on laptop computer with headphones. In order to avoid a learning effect of performing these cognitive function tests, participants will be required to perform the battery of tests and repeat it three times during the habitual diet screening week. Cognitive tests will include: 1) Groton Maze learning task (to assess executive function), 2) Identification test (to assess attention), 3) Psychomotor vigilance task (to assess reaction times) 4) One card learning task (to assess visual learning) 5) two-back test (to assess working memory). This battery of tests will be done during the habitual dietary screening week, as well as before and after each dietary intervention.

*Blood sampling and collection*

After an overnight fast a venous blood sample of ten millilitres will be drawn by venous puncture from the brachial vein of the participant for the assessment of biomarkers of cardiovascular and metabolic health. These biomarkers include lipid profiles (concentrations of triglycerides, LDL-, HDL- and total cholesterol), interleukin-6 (IL-6), interleukin-1 (IL-1), high-sensitivity C-reactive protein (hsCRP), tumour necrosis factor alpha (TNF-α), ferritin, serum amyloid P. The blood sample will also be used to assess thyroid function by measuring levels of thyroid stimulating hormone (TSH), triiodothyronine (T3) and thyroxine (T4). Blood samples will be taken before and after both dietary interventions.

*Basal metabolic rate*

Basal metabolic rate will be measured while participants are lying supine on a plinth with a face mask covering their mouth and nose. The face mask will be attached to a respiratory gas analyser. Oxygen consumption, carbon dioxide production, ventilation and respiratory rate will be recorded throughout the procedure using a computerized metabolic system (Quark ergo, COSMED, Rome, Italy). Participants will lie supine for a period of 15 minutes after which the average oxygen consumption of the last 5 minutes will be recorded as BMR. Average energy expenditure (kcal/min/kg) over the last five minutes will then be recorded as BMR. The room temperature of the laboratory will be maintained at 27 degrees Celsius for all participants.

**3.6 Data analysis**

Data analysis will be done using IBM SPSS Statistics (version 24, IBM Corporation, NY, USA). A two-tailed statistical significance will be accepted at P < 0.05. Each outcome variable assessed will be analysed according to diet (KD or HCLF) and time (before and after each diet) using a repeated measures two-way ANOVA. Where appropriate, a Student-Newman-Keuls post-hoc test will be used to assess the origin of any significant differences detected by the ANOVA models. Variables will be adjusted for potential confounders which will be determined using multiple linear regressions. A power analysis has indicated that total of 20 participants would provide 85% power to obtain a medium effect size for BMR (Cohen’s d effect size = 0.3) [[40](#_ENREF_40)].

1. **Funding:**

This study will be funded in part from the Exercise Physiology Laboratory and Brain Function Research Group (BFRG) budget and in addition external sources will be approached.

| Consumables | Total cost |
| --- | --- |
| Consumables (printing of questionnaires, hypertonic saline, needles, anaesthetic cream) | R 16000.00 |
| Freestyle Optimum β-Ketone monitor | R 2200.00 |
| Freestyle Optimum β-Ketone strips | R 2900.00 |
| BD Vacutainer tubes | R 1000.00 |
| Assay kits for hsCRP, IL-1, IL-6, TNF-α, ferritin, SAP, TSH, T_3_, T_4_ | R 40 000.00 |
| Cognitive function tests | R 30 000.00 |

1. **Problems:**

There may be some difficultly in recruiting enough participants due to the length and demanding requirements of the study. It is also foreseen that some individuals will be unwilling to participant due to the invasive method used to induce experimental pain as well as the pain itself. To ensure a big enough sample size recruitment will take place at sports clubs across Johannesburg and at the University of the Witwatersrand. Participants will receive weekly feedback and reminders throughout the intervention period in attempt to increase subject dietary compliance.

1. **References:**

1. Noakes, T.D., et al., *The Real Meal Revolution: Changing the world, one meal at a time* 2013, South Africa: Quivertree Publications

2. Gasior, M., M.A. Rogawski, and A.L. Hartman, *Neuroprotective and disease-modifying effects of the ketogenic diet.* Behav Pharmacol, 2006. **17**(5-6): p. 431-9.

3. Voster, H.H., J.B. Badham, and C.S. Venter, *Food-Based Dietary Guidelines for South Africa: An introduction to the revised food-based dietary guidelines for South Africa.* South African J Clin Nutr 2013. **26**(3): p. S5- S12.

4. Manninen, A.H., *Metabolic effects of the very-low-carbohydrate diets: misunderstood "villains" of human metabolism.* J Int Soc Sports Nutr, 2004. **1**(2): p. 7-11.

5. McClernon, F.J., et al., *The effects of a low-carbohydrate ketogenic diet and a low-fat diet on mood, hunger, and other self-reported symptoms.* Obesity (Silver Spring), 2007. **15**(1): p. 182-7.

6. McAuley, K.A., et al., *Comparison of high-fat and high-protein diets with a high-carbohydrate diet in insulin-resistant obese women.* Diabetologia, 2005. **48**: p. 8-16.

7. Sondike, S.B., N. Copperman, and M.S. Jacobson, *Effects of a low-carbohydrate diet on weight loss and cardiovascular risk factor in overweight adolescents.* J Pediatr, 2003. **142**(3): p. 253-8.

8. Samaha, F.F., et al., *A Low-Carbohydrate as Compared with a Low-Fat Diet in Severe Obesity.* N Engl J Med, 2003. **348**: p. 2074-2081.

9. Keogh, J.B., et al., *Effects of weight loss from a very-low-carbohydrate diet on endothelial function and markers of cardiovascular disease risk in subjects with abdominal obesity.* Am J Clin Nutr, 2008. **87**(3): p. 567-76.

10. Westman, E.C., et al., *The effect of a low-carbohydrate, ketogenic diet versus a low-glycemic index diet on glycemic control in type 2 diabetes mellitus.* Nutr Metab (Lond), 2008. **5**: p. 36.

11. Shai, I., et al., *Weight loss with a low-carbohydrate, Mediterranean, or low-fat diet.* N Engl J Med, 2008. **359**(3): p. 229-41.

12. Foster, G.D., et al., *A randomized trial of a low-carbohydrate diet for obesity.* N Engl J Med, 2003. **348**(21): p. 2082-90.

13. Meckling, K.A., C. O'Sullivan, and D. Saari, *Comparison of a low-fat diet to a low-carbohydrate diet on weight loss, body composition, and risk factors for diabetes and cardiovascular disease in free-living, overweight men and women.* J Clin Endocrinol Metab, 2004. **89**(6): p. 2717-23.

14. Stern, L., et al., *The effects of low-carbohydrate versus conventional weight loss diets in severely obese adults: one-year follow-up of a randomized trial.* Ann Intern Med, 2004. **140**(10): p. 778-85.

15. Sharman, M.J., et al., *A ketogenic diet favorably affects serum biomarkers for cardiovascular disease in normal-weight men.* J Nutr, 2002. **132**(7): p. 1879-85.

16. Noakes, M., et al., *Comparison of isocaloric very low carbohydrate/ high saturated fat and high carbohydrate/low saturated fat diets on body composition and cardiovascular risk.* Nutr Metab (Lond), 2006. **3**(7).

17. Westman, E.C., et al., *Effect of a low-carbohydrate, ketogenic diet program compared to a low-fat diet on fasting lipoprotein subclasses.* Int J Cardiol, 2006. **110**(2): p. 212-6.

18. Aude, Y.W., et al., *The national cholesterol education program diet vs a diet lower in carbohydrates and higher in protein and monounsaturated fat: a randomized trial* Arch Intern Med, 2004. **164**(19): p. 2141-6.

19. Macphail, K., *C-reactive Protein, Chronic Low Back Pain and, Diet and Lifestyle.* J Pain Relief, 2014. **3**(5).

20. Dansinger, M.L., et al., *Comparison of the Atkins, Ornish, Weight Watchers, and Zone diets for weight loss and heart disease risk reduction: a randomized trial.* JAMA, 2005. **293**(1): p. 43-53.

21. Sharman, M.J. and J.S. Volek, *Weight loss leads to reductions in inflammatory biomarkers after a very-low-carbohydrate diet and a low-fat diet in overweight men.* Clin Sci (Lond), 2004. **107**(4): p. 365-9.

22. Ruskin, D.N., M. Kawamura, and S.A. Masino, *Reduced pain and inflammation in juvenile and adult rats fed a ketogenic diet.* PLoS One, 2009. **4**(12): p. e8349.

23. Di Lorenzo, C., et al., *Short term improvements of migraine headaches during ketogenic diet: a prospective observational study in a dietician clinical setting* The Journal of Headache and Pain, 2013. **14**((Suppl 1)).

24. Di Lorenzo, C., et al., *Central mechanisms of migraine improvements with ketogenic diet: an evoked potentials study.* The Journal of Headache and Pain 2014. **15**(Suppl 1).

25. Afari, N., et al., *C-reactive protein and pain sensitivity: findings from female twins.* Ann Behav Med, 2011. **42**(2): p. 277-83.

26. Hartman, A.L., et al., *The neuropharmacology of the ketogenic diet.* Pediatr Neurol, 2007. **36**(5): p. 281-92.

27. Masino, S.A. and D.N. Ruskin, *Ketogenic diets and pain.* J Child Neurol, 2013. **28**(8): p. 993-1001.

28. Zhao, Q., et al., *Detrimental effects of the ketogenic diet on cognitive function in rats.* Pediatr Res, 2004. **55**(3): p. 498-506.

29. Greenwood, C.E. and G. Winocur, *Learning and memory impairment in rats fed a high saturated fat diet.* Behav Neural Biol, 1990. **53**(1): p. 74-87.

30. Halyburton, A.K., et al., *Low- and high-carbohydrate weight-loss diets have similar effects on mood but not cognitive performance.* Am J Clin Nutr, 2007. **86**(3): p. 580-7.

31. Holloway, C.J., et al., *A high-fat diet impairs cardiac high-energy phosphate metabolism and cognitive function in healthy human subjects.* Am J Clin Nutr, 2011. **93**(4): p. 748-55.

32. Willi, S.M., et al., *The effects of a High-protein, Low-fat, Ketogenic Diet on Adolescents With Morbid Obesity: Body Composition, Blood Chemistries, and Sleep Abnormalities.* Pediatrics, 1998. **101**(1): p. 61-67.

33. Hallbook, T., J. Lundgren, and I. Rosen, *Ketogenic diet improves sleep quality in children with therapy-resistant epilepsy.* Epilepsia, 2007. **48**(1): p. 59-65.

34. Afaghi, A., H. O'Connor, and C.M. Chow, *Acute effects of the very low carbohydrate diet on sleep indices.* Nutritional NeuroScience, 2008. **11**(4): p. 146-154.

35. Liu, R., et al., *Association between Sleep Quality and C-Reactive Protein: Results from National Health and Nutrition Examination Survey, 2005-2008.* PLoS One, 2014. **9**(3): p. E92607.

36. Smith, M.T. and J.A. Haythornthwaite, *How do sleep disturbance and chronic pain inter-relate? Insights from the longitudinal and cognitive-behavioral clinical trials literature.* Sleep Med Rev, 2004. **8**(2): p. 119-32.

37. Volek, J.S., et al., *Body composition and hormonal responses to a carbohydrate-restricted diet.* Metabolism, 2002. **51**(7): p. 864-70.

38. Fery, F., et al., *Hormonal and metabolic changes induced by an isocaloric isoproteinic ketogenic diet in healthy subjects.* Diabete Metab, 1982. **8**(4): p. 299-305.

39. Yancy, W.S., Jr., et al., *A low-carbohydrate, ketogenic diet versus a low-fat diet to treat obesity and hyperlipidemia: a randomized, controlled trial.* Ann Intern Med, 2004. **140**(10): p. 769-77.

40. Cohen, J., *A power primer.* Psychol Bull, 1992. **112**(1): p. 155-9.

41. Buysse, D.J., et al., *The Pittsburgh Sleep Quality Index: a new instrument for psychiatric practice and research.* Psychiatry Res, 1989. **28**(2): p. 193-213.

42. Goldberg, D.P., et al., *A comparison of two psychiatric screening tests.* Br J Psychiatry, 1976. **129**: p. 61-7.

43. Bull, F.C., T.S. Maslin, and T. Armstrong, *Global physical activity questionnaire (GPAQ): nine country reliability and validity study.* J Phys Act Health, 2009. **6**(6): p. 790-804.
